# Unraveling Surface Reconstruction of MOF‐Derived La, P‐Co_3_O_4_ for Energy‐Efficient Water and Urea Electrolysis

**DOI:** 10.1002/smtd.202500938

**Published:** 2025-09-04

**Authors:** Bharathi Arumugam, Pandian Mannu, Ranjith Kumar Darman, Ramkumar Vanaraj, Krishnapandi Alagumalai, Chi‐Liang Chen, Tae Hwan Oh, Chung‐Li Dong, Seong‐Cheol Kim

**Affiliations:** ^1^ School of Chemical Engineering Yeungnam University Gyeongsan 38541 South Korea; ^2^ Department of Physics Tamkang University New Taipei City 25137 Taiwan; ^3^ National Synchrotron Radiation Research Center Hsinchu 30076 Taiwan

**Keywords:** catalyst stability, co‐doped Co_3_O_4_, energy conversion, MOF‐derived catalysts, surface reconstruction, urea electrolysis, water splitting

## Abstract

Constructing robust electrocatalysts and shedding light on the processes of surface reconstruction is crucial for sustained hydrogen production and a deeper understanding of catalytic behavior. Here, a novel ZIF‐67‐derived lanthanum‐ and phosphorus‐co‐doped Co_3_O_4_ catalyst (La, P‐Co_3_O_4_) has been reported. X‐ray absorption spectroscopy (XAS) confirms that the La and P co‐doping reduces the coordination number (CN), improves oxygen vacancies (O_v_), and leads to lattice distortion. Soft XAS confirms that Co^2+^ exists predominantly in La, P‐Co_3_O_4_ than in Co_3_O_4_. Investigation of surface reconstruction with in situ Raman spectroscopy_,_ revealing that La, P‐Co_3_O_4_ reconstructs earlier into catalytically active γ‐CoOOH during the oxygen evolution reaction (OER) process. As a result, La, P‐Co_3_O_4_ exhibits commendable electrocatalytic performance with minimal overpotentials of 351 mV for the OER, 222 mV for the hydrogen evolution reaction (HER), and 1.46 V for the urea oxidation reaction (UOR) to achieve a current density of 50 mA cm^−2^. A two‐electrode electrolyzer using La, P‐Co_3_O_4_ as anode and cathode, achieving 19.4% energy savings during urea electrolysis compared to overall water electrolysis while maintaining stability for 72 h. This study provides a new perspective for understanding the mechanism and co‐doping impact on the physicochemical properties of spinel Co_3_O_4_ for sustainable energy conversion.

## Graphical abstract

Enhanced OER by surface reconstruction: The dopant promotes surface reconstruction and surface oxygen vacancies, which enhance electrochemical activity, charge transfer, and stability, resulting in improved water and urea electrolysis.

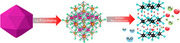

## 1 Introduction

The widespread use of synthetic fertilizers and conventional fossil fuels has contributed to global ecological and economic disparities. Advancing reusable, cost‐effective, and environmentally friendly methods is critical for addressing the substantial energy demands and pollution issues that are exacerbated by current global issues. Thus, transitioning from fossil fuel technologies to green renewable hydrogen (H_2_) energy without adverse effects on the environment requires advances in energy conversion technology. Water electrolysis effectively produces hydrogen using excess electricity, thereby decreasing the dependence on conventional fossil fuels.^[^ ^1^, ^2^ ^]^ Theoretically, a thermodynamic Gibbs free energy (ΔG) of ≈237.2 kJ mol^−1^ is required for water splitting. However, the primary obstacle in commercializing water electrolysis is the slow anodic oxygen evolution reaction (OER), which has a substantial thermodynamic potential (1.23 V), resulting from two stages of O─H bond cleavage and subsequent O─O bond formation. This process involves the transfer of four electrons and a considerable kinetic barrier. The reduced O_2_ generation rate at the anodic side and the potential explosion of H_2_/O_2_ gases increase the expenditure of the water electrolyzer. In addition, the use of limited freshwater sources as electrolytes for water splitting and the lower economic value of oxygen than hydrogen make it impractical to meet commercial energy demands.^[^ ^3^ ^]^

From this perspective, the replacement of the inefficient OER with wastewater or biomass resources (which possess strong oxidizing capabilities) using a hybrid electrocatalytic method presents a substantial opportunity to address both environmental and energy issues. The electrochemical urea oxidation reaction (UOR) is highly significant because of its substantially lower thermodynamic cell potential (0.37 V) than that of the OER (1.23 V), and it significantly improves hydrogen generation by providing a six electron process simultaneously (CO(NH_2_)_2_ + 6OH^−^ → CO_2_ + N_2_ + 5H_2_O + 6e^−^). In addition, UOR‐based fuel cells are economically viable, exhibit natural abundance, and deliver a high energy density (3000 Wh kg^−1^), providing a secure means to accomplish both objectives simultaneously.^[^ ^4^, ^5^ ^]^ Over the past few decades, researchers have investigated earth‐abundant, first‐row (3d) transition metal oxides, including 3d metal oxyhydroxides,^[^ ^6^ ^]^ oxide perovskites,^[^ ^7^, ^8^ ^]^ metal phosphate composites,^[^ ^9^ ^]^ metal borate composites,^[^ ^10^ ^]^ and molecular complexes.^[^ ^11^, ^12^ ^]^ The OER performance of bimetallic or multimetallic components comprising iron, cobalt, and nickel is promising.^[^ ^13^, ^14^ ^]^ In particular, the surface self‐reconstruction of Co sites in Co‐based oxides into Co(IV) oxyhydroxides, including di‐µ‐oxo‐bridged Co─Co sites and exhibiting oxygen vacancy (O_v_) by doping, has been demonstrated, resulting in enhanced electrocatalytic activity.^[^ ^15^, ^16^, ^17^ ^]^

Co_3_O_4_, a metallic oxide with a spinel structure, is commonly used in alkaline electrocatalytic water splitting because of its excellent electrochemical stability.^[^ ^9^, ^18^ ^]^ However, Co_3_O_4_ exhibits poor electrical conductivity, high overpotential, and limited intrinsic activity, thereby reducing its practical applicability.^[^ ^19^ ^]^ Metal–organic frameworks (MOFs) have emerged as a fast‐growing field of research in the past two decades.^[^ ^20^, ^21^, ^22^ ^]^ The primary advantage of MOFs lies in their adjustable frameworks created by modular self‐assembly reactions involving metal ions or clusters and organic ligands.^[^ ^23^, ^24^, ^25^, ^26^ ^]^ Despite their chemical instability, which often limits their direct use in OER electrocatalysis, they present numerous opportunities for chemical conversion into diverse nanostructures, thereby allowing for the retention of some advantages of MOFs.^[^ ^27^ ^]^ MOF‐derived Co_3_O_4_ is particularly promising for electrocatalysis because of its hierarchical porous structure, uniform metal distribution, intrinsic activity, scalability, and tunability. However, its practical performance is hindered by several limitations, including low electrical conductivity, insufficient active sites, limited OER kinetics, agglomeration, and structural collapse upon pyrolysis.^[^ ^28^, ^29^, ^30^, ^31^ ^]^ To address these challenges in electrocatalysis (or water electrolysis), several modification techniques have been used, such as doping, defect engineering, morphological control, surface functionalization, hybrid structure, and composite materials. Among these, doping is the most effective strategy for modulating the electronic structure, active site density, and catalytic efficiency of Co_3_O_4_.^[^ ^28^, ^31^ ^]^ Doping facilitates tuning the electronic structure tuning, enhancing O_v_ formation, and stabilizing of high‐valent Co states, all of which are critical for improving OER activity and long‐term durability. The incorporation of lanthanum and phosphorus into the Co_3_O_4_ lattice offers distinct and synergistic advantages. Because of their larger ionic radius and electron‐donating characteristics, La^3^⁺ ions enhance charge transfer, promote stable high‐valent cobalt species (Co^3^⁺/Co⁴⁺), and accelerate OER kinetics. P doping primarily generates O_v_, enhances electrolyte wettability, and alters the surface charge distribution via the formation of Co─P bonds, thereby facilitating the adsorption and desorption of oxygen intermediates. La and P co‐doping in Co_3_O_4_ produces a synergistic effect, where La enhances electrical conductivity and redox activity, and P enhances active site exposure and surface reactivity. Recently, Sieon et al. used a pulsed laser synthesis approach to synthesize Ir‐Co_3_O_4_@NC (ZIF‐67‐derived Co_3_O_4_ doped with iridium and then anchored on N‐doped carbon). Water electrolysis performed in a 1‐M KOH electrolyte required 1.62 V to obtain a current density of 10 mA cm^−2^.^[^ ^32^ ^]^ Doping ZIF‐67‐derived Co_3_O_4_ with Fe (Fe‐V_O_‐Co_3_O_4_‐ZIF/NF) enhanced the OER activity and O_v_ concentration.^[^ ^33^ ^]^ M‐doped CoP (M = Ni, Mn, Fe) hollow polyhedron frameworks (HPFs) were synthesized using ZIF‐67 as a template, followed by oxidation and phosphorization. In particular, the Ni‐CoP/HPFs exhibited a current density of −10 mA cm^−2^, an overpotential of 92 mV, and sustained activity for a minimum of 21 h.^[^ ^34^ ^]^

In this study, La‐ and P‐co‐doped ZIF‐67‐derived Co_3_O_4_ (La, P‐Co_3_O_4_) was designed as an efficient catalyst that exhibits outstanding electrocatalytic performance and requires only 351 and 222 mV for the OER and hydrogen evolution reaction (HER), respectively, at 50 mA cm^−2^. The Tafel slopes were 39.1‐ and 28.1‐mV dec^−1^ for the OER and HER, respectively, indicating rapid reaction kinetics. In addition, a two‐electrode electrolyzer using La, P‐Co_3_O_4_ as both the anode and cathode achieved voltages of 1.63 and 1.44 V for water and urea electrolysis, respectively, demonstrating remarkable durability over 72 h at a current density of 10 mA cm^−2^. The catalytic activity was synergistically enhanced by developing a hollow conductive matrix with superior electrical properties ascribed to electronic structure modification, O_v_, which provides a higher active site density. X‐ray absorption spectroscopy was employed to confirm the co‐doping, O_v_, catalyst coordination number, oxidation state, and spin state of cobalt. In addition, in situ/operando Raman results demonstrate the dynamic surface evolution of defective electrocatalysts and reveal the active species involved in the electrocatalytic process. This study presents an innovative method for fabricating high‐efficiency, cost‐effective electrocatalysts and its structural evolution during electrocatalysis.

## 2 Results and Discussion

### 2.1 Structural and Morphological Characterization

**Figure 1:**
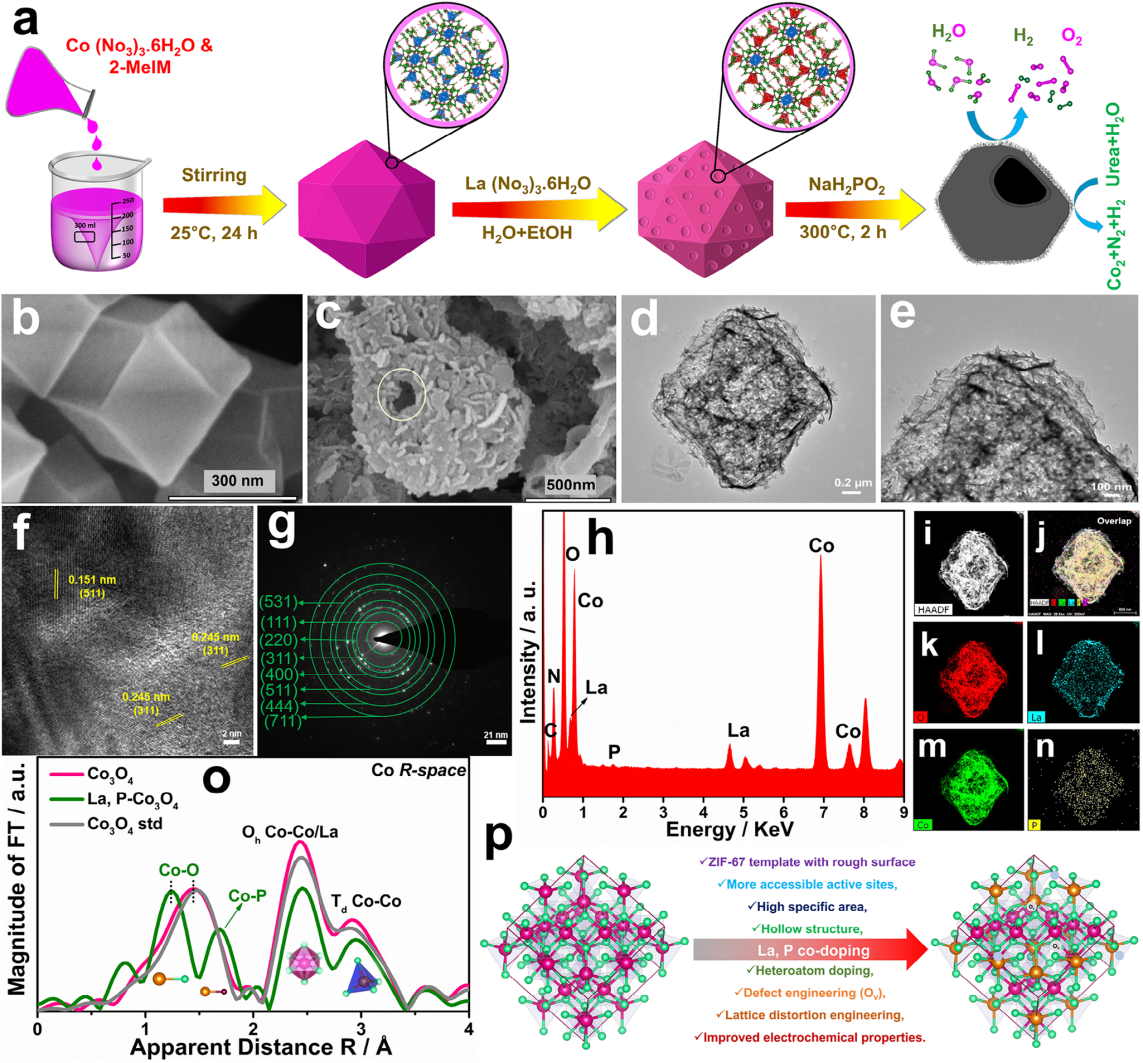
a) Schematic of the synthesis of La, P‐Co_3_O_4_. FESEM images of as‐synthesized b) ZIF‐67, c), and La, P‐Co_3_O_4_. d–f) HRTEM images of La, P‐Co_3_O_4_. g) SAED pattern, h) elemental spectra, i–n) HAADF‐STEM image, and EDS mapping of La, P‐Co_3_O_4_. o) Fourier transform of k^3^‐weighted Co K‐edge EXAFS spectra, and p) schematic of the merits of La and P co‐doping.

**Figure** **1**a illustrates the synthetic strategy used to synthesize the La, P‐Co_3_O_4_ electrocatalyst. FESEM images of ZIF‐67, Co_3_O_4_, La‐ZIF‐67, La‐Co_3_O_4_, P‐Co_3_O_4_, and La, P‐Co_3_O_4_ are shown in Figure 1b,c and Figure S1 (Supporting Information). As shown in Figure S1a (Supporting Information), the ZIF‐67 sample exhibits a consistent polyhedral morphology. In addition, a La (NO_3_)_3_·6H_2_O precursor was reacted with ZIF‐67 to form La‐doped ZIF‐67 via a cationic etching process. Irrespective of La doping, the polyhedral morphology of the parent ZIF‐67 was retained (Figure S1b, Supporting Information). During the reaction, the chelating agent 2‐MIM promoted the protonation process with the formation of OH^−^ ions. Consequently, the produced OH^−^ ions were coprecipitated to form dissociative Co^2^⁺ and La^3^⁺ ions.^[^ ^35^ ^]^ Furthermore, phosphorization was performed to yield a La‐ and P‐co‐doped Co_3_O_4_ electrocatalyst (Figure 1c; Figure S1f, Supporting Information). Similarly, the original polyhedral structure was retained in the as‐prepared La, P‐Co_3_O_4_ catalyst, which was characterized by the growth of 2D nanosheets on its surface (Figure 1c–e). The calcination process of ZIF‐67 and La‐ZIF‐67 yielded Co_3_O_4_ and La‐Co_3_O_4_, which induced the breakdown of the 3D framework (Figure S1c,e, Supporting Information). The La, P‐Co_3_O_4_ catalyst exhibited no structural degradation, highlighting the stability imparted by phosphorus doping (Figure S1d,f, Supporting Information).^[^ ^36^, ^37^ ^]^ Figure 1d,e shows the HRTEM images, validating that the La, P‐Co_3_O_4_ electrocatalyst preserves its 3D framework with a textured surface regardless of the doping and pyrolysis processes. The HRTEM d‐spacing values of 0.243 and 0.245 nm represent pristine Co_3_O_4_ and La, P‐Co_3_O_4_, respectively, corresponding to the (311) plane of the spinel crystal structure (Figure 1f). In the selected area electron diffraction (SAED) pattern for Co_3_O_4_ (Figure 1g), the obtained diffraction rings correspond to the (111), (220), (311), (400), (511), (531), (444), and (711) planes of the spinel crystal structure. Similarly, the elemental composition of the La, P‐Co_3_O_4_ electrocatalyst was characterized using high‐angle annular dark‐field scanning transmission electron microscopy (HAADF‐STEM) and energy‐dispersive X‐ray spectroscopy (EDS), which confirmed the uniform distribution of Co, O, La, and P (Figure 1h–n). In addition, the atomic‐level elemental mapping of La and P provided additional evidence of successful doping (Figure 1i,n). Furthermore, the extended X‐ray absorption fine structure (EXAFS) technique was used to explore the electronic and geometric structures of the catalysts. As shown in Figure 1o, the k^3^‐weighted k‐edge EXAFS spectra for Co_3_O_4_ show three main signals at 1.44, 2.43, and 2.92 Å, which are related to the Co─O, Co─Co_Oh_ (octahedral), and Co─Co_Td_ (tetrahedral) scattering pathways, respectively. However, the additional peaks at 1.69 Å (Co─P) and 2.46 Å in La, P‐Co_3_O_4_ confirm the co‐doping of La and P. The decrease in the Co─O coordination number compared to pristine Co_3_O_4_ indicates electronic alterations in Co_3_O_4_ due to La and P co‐doping. In addition, the general advantages of the co‐doping of Co_3_O_4_ with La and P are illustrated in Figure 1p.

**Figure 2:**
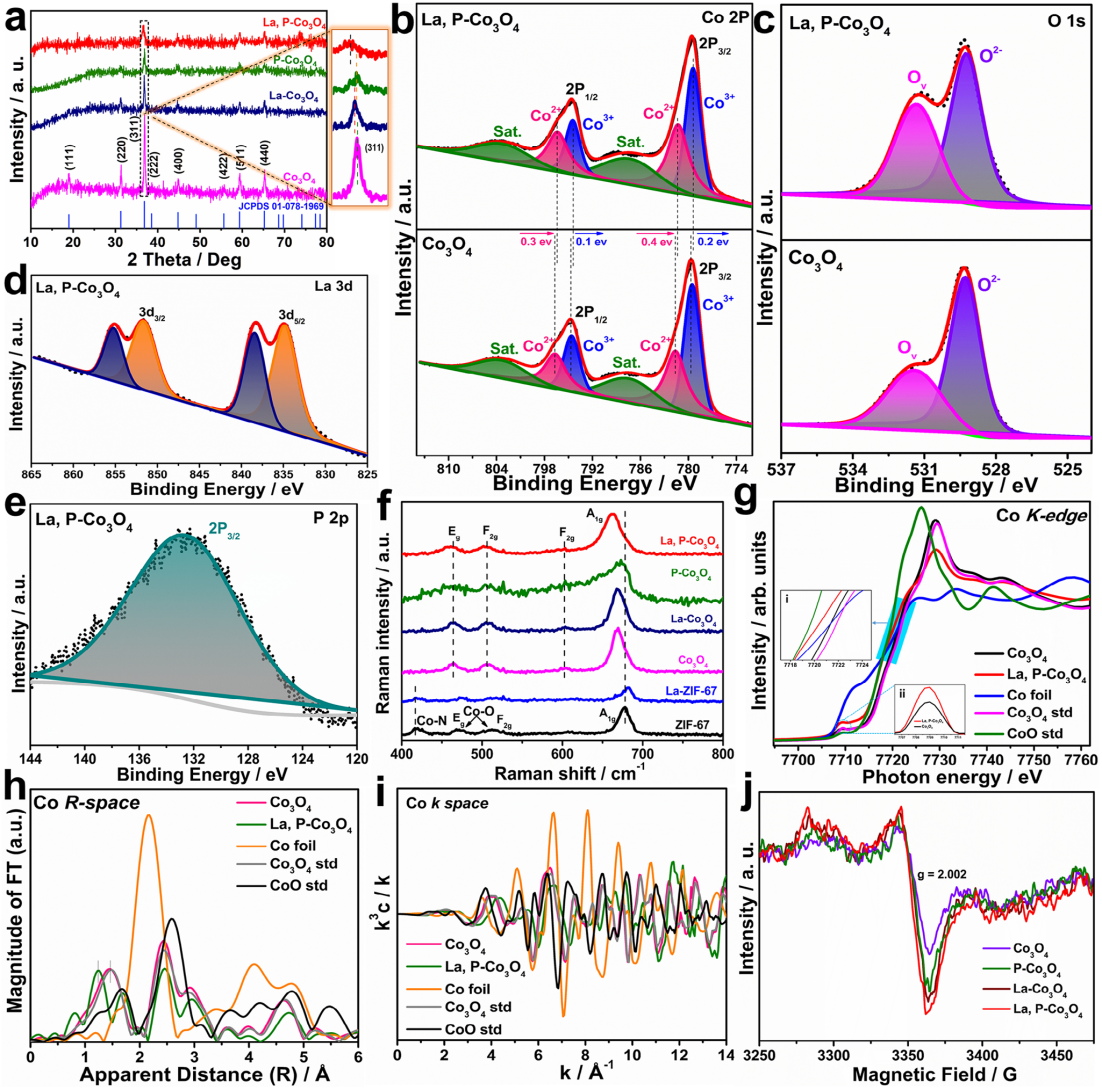
a) PXRD patterns of Co_3_O_4_, La‐Co_3_O_4_, P‐Co_3_O_4_, and La, P‐Co_3_O_4_. The magnified figure shows the (311) plane corresponding to the PXRD patterns. XPS spectra of both Co_3_O_4_ and La, P‐Co_3_O_4_: b) Co, c) O, d) La, and e) P (La, P‐Co_3_O_4_). f) Raman spectra of all catalysts. g) Co K‐edge XANES spectra of Co_3_O_4_ and La, P‐Co_3_O_4_. h) Fourier transforms of k^3^‐weighted Co K‐edge EXAFS. i) Fourier transform k^3^𝜒 data for EXAFS oscillations Co k‐space spectra of Co_3_O_4_ and La, P‐Co_3_O_4_. j) ESR spectra of all Co_3_O_4_‐based catalysts.

The crystalline structures of the as‐prepared electrocatalysts were investigated using X‐ray diffraction (XRD) (**Figure** **2**a). The XRD pattern of ZIF‐67 matches the crystal planes of previously reported ZIF‐67 (Figure S2, Supporting Information).^[^ ^38^, ^39^ ^]^ In La‐Co_3_O_4_, the peak intensity is reduced compared to that of pristine Co_3_O_4_, indicating the effective incorporation of La into the framework, further resulting in point defects or structural disorder.^[^ ^40^ ^]^ In addition, a lower shift in θ at 36.8° was due to the lattice expansion of the larger La (1.16 Å), confirming the successful La doping in Co_3_O_4_ (magnified image in Figure 2a).^[^ ^41^ ^]^ The ZIF‐67‐derived Co_3_O_4_ was characterized by distinctive diffraction peaks (JCPDS 01‐078‐1969).^[^ ^42^, ^43^ ^]^ No new peaks appeared after phosphorus doping because of the lower concentration of P. The co‐doping process creates strain, which disturbs the Co^3^⁺/Co^2^⁺ redox equilibrium in La, P‐Co_3_O_4_, thereby stabilizing Co⁴⁺ species, which is highly beneficial in enhancing electrocatalytic activity.^[^ ^41^ ^]^ Raman analysis was performed to obtain structural, chemical, and vibrational insights into the catalysts (Figure 2f). The Raman peaks at 469 cm^−1^ (E_g_), 512 cm^−1^ (F_2g_), and 677 cm^−1^ (A_1g_) signify the ZIF‐67, whereas the strength of Co─O bonds is further intensified for the ZIF‐67‐derived Co_3_O_4_. In the La‐ZIF‐67 catalyst, blue shift peaks appeared at 677–681 cm^−1,^ indicating alterations in the C─N bond. Because of the calcination of ZIF‐67‐derived Co_3_O_4_, the Co─N peak at 417 cm^−1^ disappears. In addition, the Co_3_O_4_‐based catalysts exhibit distinct spinel structural modes, with the A_1g_ peak at 684 cm^−1^ corresponding to Co^3^⁺─O stretching and the F_2g_ peak at 520 cm^−1^ associated with Co^2^⁺─O.

X‐ray photoelectron spectroscopy (XPS) was used to examine the surface compositions and chemical states of the catalysts (Figure 2b–e). The XPS survey spectra confirmed the presence of Co, O, La, and P in Co_3_O_4_ and La, P‐Co_3_O_4_, demonstrating the purity of the as‐prepared catalyst, which was free from contamination (Figure S3, Supporting Information). As shown in Figure 2b, the Co 2p spectra of ZIF‐67‐derived Co_3_O_4_ show two peaks at 779.6 eV (Co 2p_3__/__2_) and 794.7 eV (Co 2p₁_/__2_), accompanied by satellite peaks at 781 and 796 eV, respectively, indicating mixed Co^2^⁺/Co^3^⁺ valence states. In addition, the binding energy difference of ΔE = 15.1 eV is consistent with the spinel structure of Co_3_O_4_, validating the presence of Co^2^⁺ and Co^3^⁺ in octahedral and tetrahedral coordination, respectively. After the co‐doping of La and P in ZIF‐67‐derived Co_3_O_4_, a negative binding energy shift of Co^2^⁺ (0.4 eV for 2p_3__/__2_ and 0.3 eV for 2p₁_/__2_) and Co^3^⁺ (0.2 eV for 2p_3__/__2_ and 0.1 eV for 2p₁_/__2_) was observed, signifying electronic alterations in the Co_3_O_4_ structure attributable to La^3^⁺ and P⁵⁺ doping. The doping stabilizes the active cobalt sites and enhances O_v_, as evidenced by the O 1s spectra (Figure 2c).^[^ ^44^ ^]^ The O 1s spectra of Co_3_O_4_ show two peaks at 529.3 eV (lattice oxygen) and 531.4 eV (O_v_). In La, P‐Co_3_O_4_, the intense O_v_ peak represents defect formation owing to the co‐doping of La and P, which enhances catalytic activity via enhanced oxygen mobility and intermediate stabilization. Furthermore, the existence of O_v_ enhances electron transport between Co^2^⁺ and Co^3^⁺ ions, thereby increasing the number of electron carriers, accelerating charge transfer, and improving electrical conductivity (Figure 2c). The high‐resolution La 3d peaks at 834.9 and 851.7 eV (Figure 2d) and the P 2p peak at 132.8 eV (Figure 2e) indicate the existence of La^3^⁺ and phosphate species in the La, P‐Co_3_O_4_ electrocatalyst. To further explore the local atomic and electronic configurations of pristine Co_3_O_4_ and La, P‐Co_3_O_4_, synchrotron X‐ray absorption near edge structure (XANES) and Extended X‐ray absorption fine structure (EXAFS) analyses were performed. (Figure 2g–i) The Co K‐edge XANES spectra (Figure 2g) reveal three primary characteristic peaks of Co_3_O_4__._ The pre‐peak is typically linked to the 1s–3d electric dipole‐forbidden transition, which gains allowance in the framework of tetrahedral symmetry. The shoulder feature observed at ≈7723 eV resulted from the charge transfer of the ligand to the metal. In addition, the prominent peak observed at 7730 eV resulted from the electric dipole transition from the 1s to 4p states. The energy position of the primary absorption peak at ≈7730 eV, along with the spectral profiles of the Co K‐edge XANES for Co_3_O_4_, La, and P‐Co_3_O_4_, closely resemble those of standard Co_3_O_4_. In contrast, they differ from the Co foil (Co in zero state) or Co─O (Co in +2 state). This observation suggests that the average Co oxidation state of Co_3_O_4_ is between +2 and +3. La, P‐Co_3_O_4_ exhibits a lower intensity at the primary absorption peak, which may be attributed to a decreased oxidation state in La, P‐Co_3_O_4_ than in Co_3_O_4_. This is due to the relative electronegativity levels, where La donates electrons to Co and P occupies the oxygen site. In addition, the electron localized at the Co site can potentially migrate to P albeit less readily than to O.^[^ ^45^ ^]^ Furthermore, the absorption edge positions (at ≈7720 eV) in La, P‐Co_3_O_4_ also display a reduced energy shift (inset (i), Figure 2g), indicating a decrease in the Co valence state. O_v_ in metal oxides generally results in two surplus electrons that migrate to adjacent metal ions, thereby diminishing their valence states. The pre‐edge peaks of the Co_3_O_4_ spinel (≈7710 eV) were caused by the dipole‐forbidden 1s → 3d transitions that combined with the Co 4p states. This result provides information about the 3d electron occupancy and local symmetry of the catalysts. The increased pre‐edge intensity (inset (ii) in Figure 2g), followed by La and P co‐doping, indicates an increased Co^2^⁺ concentration attributable to O_v_, which is also consistent with the XPS results. (Figure 2c).

The Fourier transforms of the k^3^‐weighted Co K‐edge EXAFS spectra for both the Co_3_O_4_ and La, P‐Co_3_O_4_ catalysts show three principal signals corresponding to the Co─O, Co─Co_oh_ (octahedral site), and Co─Co_Td_ (tetrahedral site) scattering pathways (Figure 2h). The reduced peak intensity of Co─O and Co─Co_oh_ bonds in La, P‐Co_3_O_4_ can be associated with the reduced coordination numbers in the Co─O of La, P‐Co_3_O_4_ compared with those in pristine Co_3_O_4_. Although Co^3+^ is reduced to Co^2+^ after the co‐doping process, the Co─O CN decreases in La, P‐Co_3_O_4_ (apparent distance from 1.44 to 1.24 Å) because of the incidence of uncoordinated Co ions caused by more O_v_ that evolved after the co‐doping process.^[^ ^46^ ^]^ In addition, the new signal at 1.69 Å is attributed to Co─P.^[^ ^47^, ^48^ ^]^ The results indicate O_v_ formation and that electronic modification occurs in Co_3_O_4_ after doping, which is consistent with the XPS profiles. To elucidate the observed alteration in the Co─O bond distance and the appearance of the Co–P peak, it is crucial to investigate the electrical and structural changes prompted by O_v_. The generated O_v_ can induce localized electron density redistribution, thereby influencing the cobalt oxidation states and the coordination environments. Thus, the reduction in the Co─O bond length results in enhanced Coulomb interactions at Co^2+^ sites. In addition, the Co–P peak at 1.69 Å indicates direct bonding between Co and P, suggesting changes in local geometry resulting from P inclusion. The appearance of this peak aligns with previously documented structural alterations resulting from heteroatom doping in transition metal oxides.^[^ ^47^, ^49^ ^]^ To further examine the atomic structure of the catalyst, the k^3^𝜒 data for the EXAFS oscillations of the Co k‐space are denoted in Figure 2i. Co_3_O_4_ and La, P‐Co_3_O_4_ exhibit dissimilar EXAFS oscillations (Figure S10, Supporting Information), suggesting varying coordination geometries surrounding the Co sites. A decrease in the oscillation amplitude of La, P‐Co_3_O_4_ compared to Co_3_O_4_ indicates more disordered octahedral sites. The disordered octahedra in La, P‐Co_3_O_4_ correspond to the pre‐edge feature of the Co K‐edge, because the intensity of the pre‐edge region is marginally greater than that of Co_3_O_4_, thereby validating the disordered coordination. The enhanced O_v_ in La, P‐Co_3_O_4_ corresponds to the pre‐edge feature of the Co K‐edge, exhibiting a higher‐intensity pre‐edge peak for La, P‐Co_3_O_4_ than for pristine Co_3_O_4_ (as shown in Figure 2f (inset (ii))).

Soft X‐ray absorption spectra of the synthesized catalysts were recorded to examine their local geometric structures and valence states. Figure S4b (Supporting Information) shows the Co L‐edge spectra of the Co_3_O_4_ and La, P‐Co_3_O_4_ catalysts. The L_3,2_‐edge spectra of transition metals are significantly influenced by hybridization with O 2p ligands, local crystal fields, and multiple structures, making them exhibiting high sensitivity to electronic structures and spin states. As a result of orbital splitting, the Co L‐edge spectra include the L_3_ and L_2_ areas at ≈779 and 794 eV, respectively. The absorption peaks at ≈777.6 and 779.8 eV correspond to the high‐spin (HS) Co^2+^ and low‐spin (LS) Co^3+^ states, respectively.^[^ ^50^ ^]^ The minor peak at 783.0 eV is linked to metal‐to‐ligand charge transfer. The relative intensity of the peak at ≈779.6 eV for La, P‐Co_3_O_4_, exceeds that of Co_3_O_4_ (Figure S4, Supporting Information), indicating the predominance of the HS Co^2+^ state due to O_v_, which supports previous reports on Co_3_O_4_.^[^ ^44^ ^]^ In addition, the deconvoluted Co‐L_3_ edge spectra show two principal features: a and b for Co_3_O_4_, and a1 and b1 for La, P‐Co_3_O_4_ (Figure S4b,c, Supporting Information). Features a and b are linked to the HS Co^2+^ (t_2g_^4^ e_g_^2^) and LS Co^3+^ (t_2g_^5^ e_g_^0^) states in octahedral coordination.^[^ ^51^, ^52^, ^53^, ^54^ ^]^ A higher a_1_/b_1_ ratio was detected in La, P‐Co_3_O_4_ than in Co_3_O_4_, indicating the existence of numerous Co^2+^. Similarly, the elevated intensity of feature a_1_ in La, P‐Co_3_O_4_ indicates the predominance of the HS Co^2+^ state (t_2g_^4^ e_g_^2^) (Figure S4c, Supporting Information). These results indicate O_v_ enrichment after La and P co‐doping, thereby enhancing the electrochemical activity. In addition, the formation of oxygen vacancies (O_v_) upon La and P co‐doping was further confirmed by electron paramagnetic resonance (EPR) spectroscopy. Both pristine and doped Co_3_O_4_ samples exhibited characteristic signals around g ≈ 2.003, attributed to unpaired electrons localized at oxygen‐deficient sites (Figure 2j). Notably, the intensity of this signal increased significantly in the La, P‐Co_3_O_4_ sample, indicating a higher concentration of O_v_. This result is consistent with the XPS analysis of the O 1s region revealing a significant increase in surface oxygen vacancies after La and P co‐doping (Figure 2c). The oxygen defect component in the O 1s spectrum increased from 41% (pristine) to 59% (La, P‐Co_3_O_4_). Together, these findings confirm that dual doping effectively promotes oxygen vacancy formation, which primarily promotes its electrical characteristics and catalytic efficiency.

### 2.2 Electrocatalytic Water Electrolysis (OER/HER) Performance

**Figure 3:**
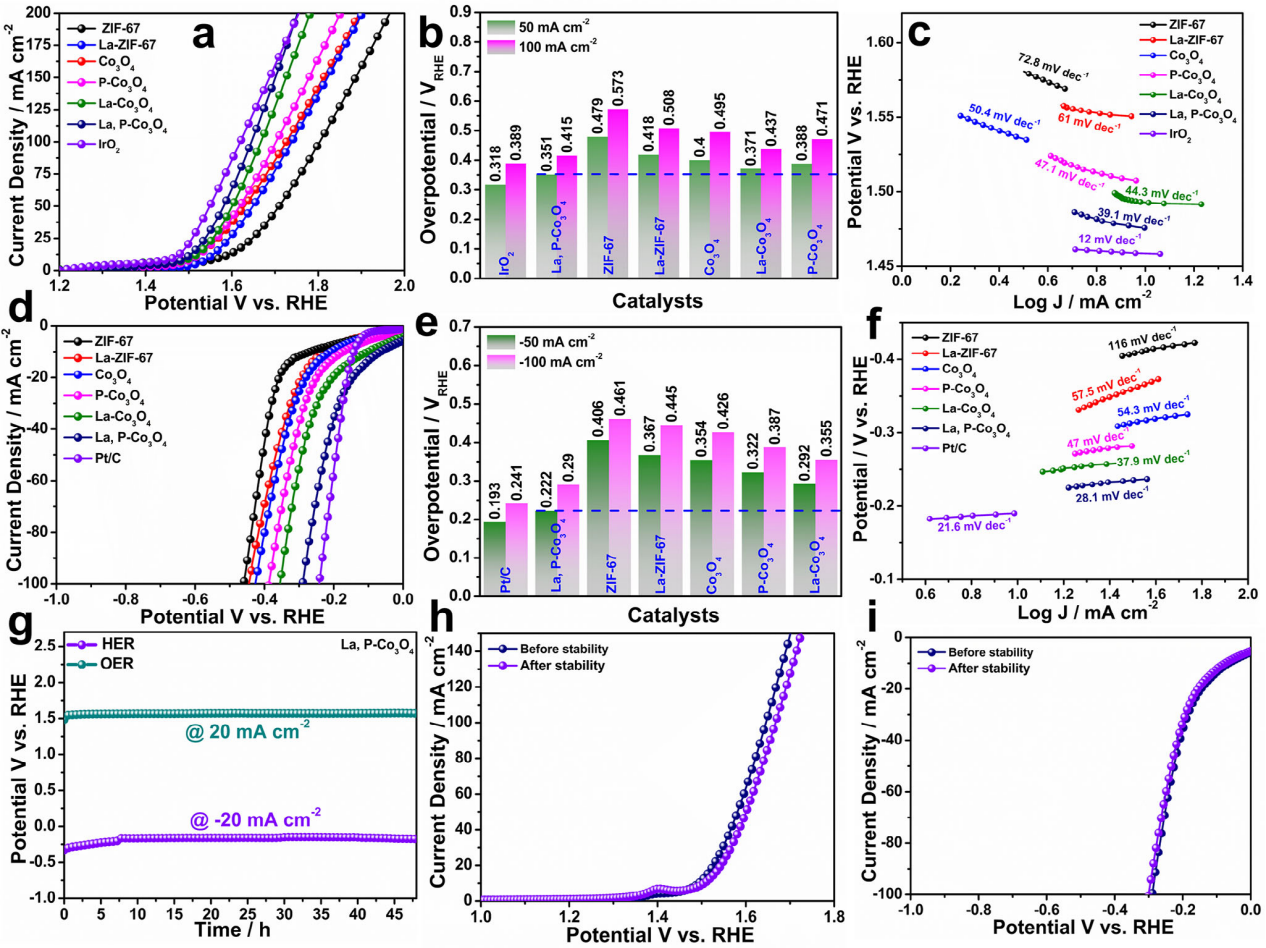
Electrochemical properties of synthesized electrocatalysts toward OER/HER activities. a) LSV polarization curve, b) overpotential, and c) Tafel plot for OER. d) LSV polarization curve, e) overpotential, and f) Tafel plot for HER. g) CP results for La, P‐Co_3_O_4_ at 20 and −20 mA cm^−2^. h,i) LSV curves obtained before and after stability test of La, P‐Co_3_O_4_ catalyst.

The electrocatalytic OER/HER activities were examined using a three‐electrode setup in a 1‐M KOH electrolyte. The LSV polarization curves for the ZIF‐67, Co_3_O_4_, La‐ZIF, La‐Co_3_O_4_, P‐Co_3_O_4_, and commercial IrO_2_ catalysts were obtained at a scan rate of 5 mV s^−1^ (**Figure** **3**a). The La, P‐Co_3_O_4_ catalyst exhibits better electrocatalytic OER activity with a lower overpotential (351 mV) than the ZIF‐67 (479 mV), La‐ZIF‐67 (418 mV), Co_3_O_4_ (400 mV), P‐Co_3_O_4_ (388 mV), and La‐Co_3_O_4_ (371 mV) catalysts at 50 mA cm^−2^ (Figure 3b). In general, Tafel plots were measured to understand the reaction kinetics of the as‐prepared catalysts. The as‐prepared La, P‐Co_3_O_4_ catalyst exhibited a smaller Tafel slope (39.1 mV dec^−1^) (Figure 3c) than the ZIF‐67 (72.8 mV dec^−1^), La‐ZIF‐67 (61 mV dec^−1^), Co_3_O_4_ (50.4 mV dec^−1^), P‐Co_3_O_4_ (47.1 mV dec^−1^), La‐Co_3_O_4_ (44.3 mV dec^−1^), and commercial IrO_2_ (12 mV dec^−1^) catalysts. Thus, the rapid increase in the current density of La, P‐Co_3_O_4,_ and its lowest overpotential among the other catalysts emphasizes the significance of the co‐doping of La and P, which is valuable for enriched electrocatalytic activity. In addition, the LSV curves of the electrocatalytic HER performance of the as‐prepared catalysts were studied in a 1‐M KOH alkaline electrolyte. The results are presented in Figure 3d. The La, P‐Co_3_O_4_ catalyst exhibited the highest catalytic activity toward HER with the lower overpotential (222 mV) compared to the ZIF‐67 (406 mV), La‐ZIF‐67 (367 mV), Co_3_O_4_ (354 mV), P‐Co_3_O_4_ (322 mV), and La‐Co_3_O_4_ (292 mV) catalysts at −50 mA cm^−2^ (Figure 3e). In addition, the reaction kinetics for the HER were analyzed with the Tafel slope derived from the steady‐state polarization curves (Figure 3f). Similarly, La, P‐Co_3_O_4_ obtained a lower Tafel slope (28.1 mV dec^−1^) than ZIF‐67 (116 mV dec^−1^), La‐ZIF‐67 (57.5 mV dec^−1^), Co_3_O_4_ (54.3 mV dec^−1^), P‐Co_3_O_4_ (47 mV dec^−1^), La‐Co_3_O_4_ (37.9 mV dec^−1^), and commercial Pt/C (21.6 mV dec^−1^), confirming its best activity toward HER. Notably, the as‐synthesized La, P‐codoped Co_3_O_4_ catalyst exhibits superior electrocatalytic activity and faster kinetics toward both the HER and OER compared to other Co_3_O_4_‐based catalysts, as demonstrated in Tables S1 and S2 (Supporting Information). This enhancement is evident from the lower overpotentials and smaller Tafel slopes achieved by the La, P‐Co_3_O_4_ catalyst, highlighting the effectiveness of the dual‐doping strategy in boosting catalytic performance. Electrochemical impedance spectroscopy (EIS) analysis demonstrates that the La, P‐Co_3_O_4_ exhibits the lowest polarization resistance among all investigated electrocatalysts (Figure S5a, Supporting Information), indicating enhanced electron transfer rates and accelerated electrocatalytic kinetics.

To accurately assess the density of active sites in La, P‐Co_3_O_4_, the electrochemical surface area (ECSA) values of the catalysts were determined by cyclic voltammetry (CV) at various scan rates in the non‐Faradaic region (Figures S6 and S7, Supporting Information). As shown in Figure S6 (Supporting Information), for the OER, La, P‐Co_3_O_4_ exhibits a superior double‐layer capacitance (C_dl_) of 26.7 mF cm^−2^ and an ECSA of 0.66 cm^2^ compared to ZIF‐67 (6.08 mF cm^−2^ and 0.152 cm^2^), La‐ZIF‐67 (15.4 mF cm^−2^ and 0.385 cm^2^), Co_3_O_4_ (17.9 mF cm^−2^ and 0.447 cm^2^), P‐Co_3_O_4_ (24.2 mF cm^−2^ and 0.605 cm^2^), and La‐Co_3_O_4_ (24.4 mF cm^−2^ and 0.61 cm^2^). Similarly, for the HER, La, P‐Co_3_O_4_ exhibits excellent C_dl_ (9.6 mF cm^−2^) and ECSA (0.24 cm^2^) compared to the other catalysts: ZIF‐67 (2.1 mF cm^−2^ and 0.05 cm^2^), La‐ZIF‐67 (2.8 mF cm^−2^ and 0.07 cm^2^), Co_3_O_4_ (3.85 mF cm^−2^ and 0.096 cm^2^), P‐Co_3_O_4_ (4.4 mF cm^−2^ and 0.11 cm^2^), and La‐Co_3_O_4_ (6.3 mF cm^−2^ and 0.15 cm^2^) (Figure S7, Supporting Information). The improved ECSA can be attributed to abundant active sites and enhanced conductivity resulting from efficient La and P co‐doping in Co_3_O_4_. This technique considerably increased the reaction kinetics of the La, P‐Co_3_O_4_. In addition, the practical application of the electrocatalysts was evaluated by their long‐term stability performance. Figure 3g shows the chronopotentiometry (CP) tests of the La, P‐Co_3_O_4_ catalyst over continuous operation for 48 h at 20 mA cm^−2^ toward both OER/HER. The obtained CP curves demonstrate the exceptional durability performance of the optimal La, P‐Co_3_O_4_ electrocatalyst. In addition, the before‐and after‐stability LSV polarization of the La, P‐Co_3_O_4_ catalyst shows a negligible potential difference, thereby confirming its superior stability performance toward the OER and HER (Figure 3h,i). The structural and chemical stability of the La, P‐Co_3_O_4_ catalyst after extended electrochemical operations (48 h of long‐term stability testing) was assessed using XPS (Figure S11, Supporting Information). The post‐catalysis XPS spectra confirm the preservation of the essential elements (Co, O, La, and P) on the catalyst surface and show no notable signal loss, which suggests minimal leaching. The post‐catalysis O 1s XPS profile reveals an enhanced intensity of the O_v_ peak compared to the lattice oxygen (O_Lattice_) and hydroxyl/adsorbed water (O_H2O_) peaks, suggesting an increase in the concentration of O_v_ throughout the reaction. The La 3d and P 2p spectra indicate strong anchoring of the dopant and a robust structure under electrochemical conditions. This result indicates dynamic surface transformation during operation, which is probably facilitated by the co‐doping of La and P. The increased defect density can further promote active site exposure and charge transfer, thereby maintaining the high activity of the catalyst over extended periods.

Therefore, the enhanced HER/OER activities of the optimal La, P‐Co_3_O_4_ catalyst were achieved via co‐doping, which simultaneously altered various physicochemical properties, including defect engineering (O_v_), surface active sites, increased surface area, crystal structure (lattice strain), and electrical conductivity. In particular, the numerous O_v_ in La and P‐Co_3_O_4_ are essential for modifying the electronic structure and improving the catalytic efficiency of the transition metal oxides during electrochemical energy conversion processes. In the OER, O_v_ serve as electron‐rich centers that enhance OH^−^ adsorption, facilitate the generation of intermediates (O, OH, OOH), and promote dynamic metal redox cycling (e.g., Co^3^⁺/Co^2^⁺), thereby reducing overpotential and accelerating O_2_ evolution. Furthermore, O_v_ generate midgap states, thereby improving electrical conductivity, optimizing the charge transfer kinetics, and enhancing overall electrocatalytic (OER/HER) performance.

### 2.3 UOR Electrolysis

**Figure 4:**
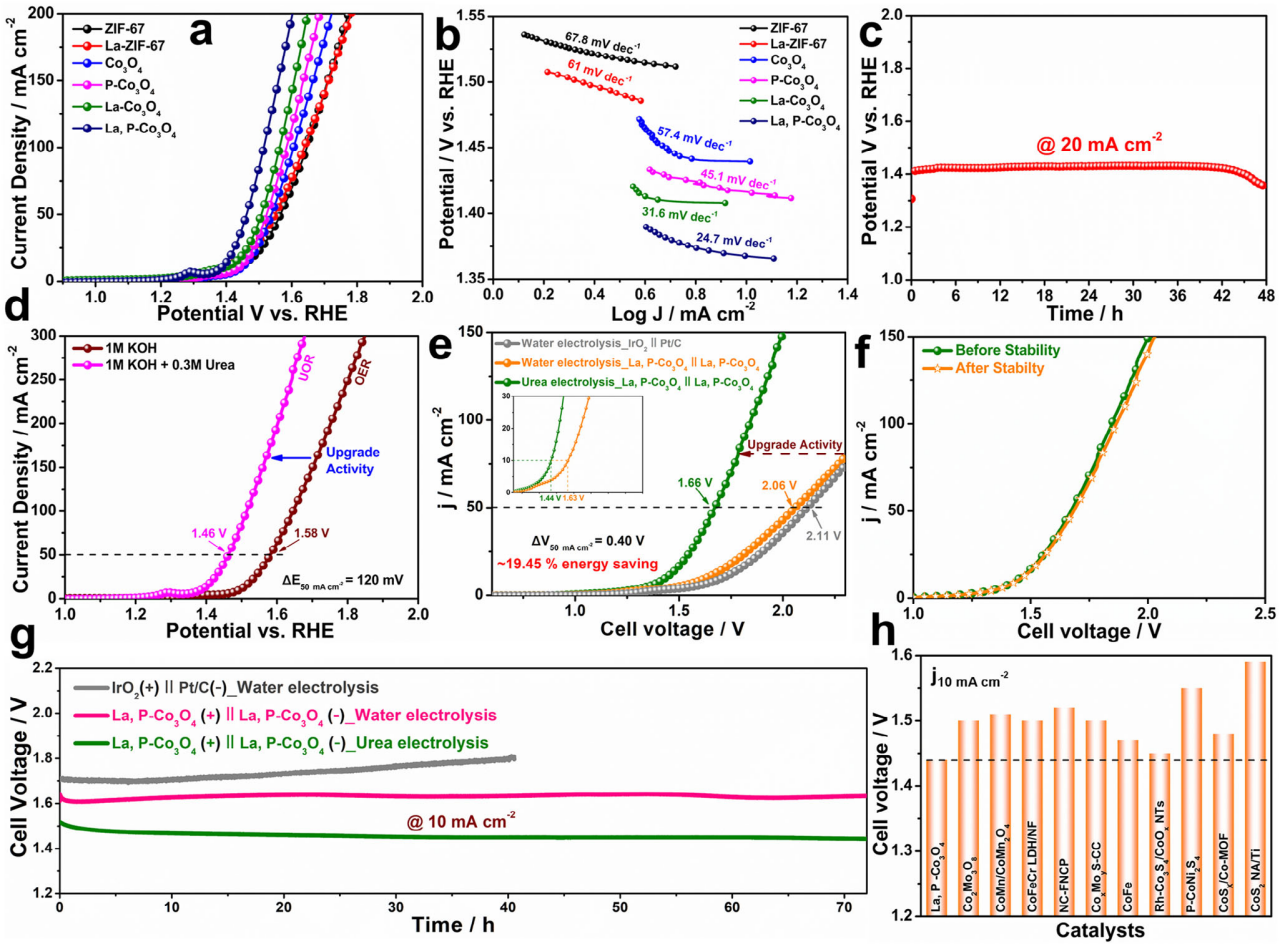
Electrochemical UOR performance: a) LSV polarization curves and b) Tafel values for ZIF‐67, La‐ZIF‐67, Co_3_O_4_, P‐Co_3_O_4_, La‐Co_3_O_4_, and La, P‐Co_3_O_4_; c) CP stability test results for La, P‐Co_3_O_4_ at 20 mA cm^−2^; d) comparison of OER/UOR curves of La, P‐Co_3_O_4_ catalyst; e) LSV polarization curve of IrO_2_||Pt/C for alkaline water electrolyzer and La, P‐Co_3_O_4_||La, P‐Co_3_O_4_ electrode for both alkaline water and urea electrolyzer; f) LSV polarization curves obtained before and after stability tests of La, P‐Co_3_O_4;_ g) CP measurement of overall water splitting, urea electrolysis for La, P‐Co_3_O_4_||La, P‐Co_3_O_4_ and IrO_2_||Pt/C electrocatalysts at 10 mA cm^−2^; h) Comparison of previously reported Co‐based electrocatalysts.

The substitution of the OER with the UOR for hydrogen production has garnered significant interest. The La, P‐Co_3_O_4_ catalyst outperforms the other catalysts in terms of the UOR, as indicated by their LSV curves. La, P‐Co_3_O_4_ exhibits a lower overpotential (1.46 V) at 50 mA cm^−2^ compared to ZIF‐67 (1.56 V), La‐ZIF‐67 (1.55 V), Co_3_O_4_ (1.53 V), P‐Co_3_O_4_ (1.52 V), and La‐Co_3_O_4_ (1.50 V) (**Figure** **4**a). The lowest Tafel value (24.7 mV dec^−1^) for La, P‐Co_3_O_4_ compared to ZIF‐67 (67.8 mV dec^−1^), La‐ZIF‐67 (61 mV dec^−1^), Co_3_O_4_ (57.4 mV dec^−1^), P‐Co_3_O_4_ (45.1 mV dec^−1^) and La‐Co_3_O_4_ (31.6 mV dec^−1^), indicates its superior chemical kinetics (Figure 4b). The minimal polarization resistance observed for La, P–Co_3_O_4_ in EIS analysis (Figure S5b, Supporting Information) indicates enhanced charge transfer efficiency and hastened reaction kinetics relative to other electrocatalysts. La, P‐Co_3_O_4_ exhibits greater C_dl_ (12.0 mF cm^−2^) and ECSA (0.3 cm^2^) than ZIF‐67 (1.7 mF cm^−2^ and 0.04 cm^2^), La‐ZIF‐67 (6.5 mF cm^−2^ and 0.16 cm^2^), Co_3_O_4_ (10.2 mF cm^−2^ and 0.255 cm^2^), P‐Co_3_O_4_ (10.3 mF cm^−2^ and 0.257 cm^2^), and La‐Co_3_O_4_ (11.5 mF cm^−2^ and 0.28 cm^2^) (Figure S8, Supporting Information). Based on the CP long‐term stability results, La, P‐Co_3_O_4_ exhibits excellent durability performance for 48 h at 20 mA cm^−2^ (Figure 4c). Figure 4d shows that the LSV polarization curve shifts to a reduced potential upon the addition of a 0.33‐M urea solution to 1‐M KOH. The obtained potential of the OER/UOR activity was 1.58/1.46 V (ΔE_50cm_^−2^ = 120 mV), respectively. The reduced anodic potential is directly proportional to the efficient generation of H_2_ at the cathode. The exceptional UOR and HER performance of La, P‐Co_3_O_4_ motivated us to advance toward practical application by developing a bifunctional catalytic urea electrolyzer for energy‐efficient H_2_ generation. Consequently, a device using La, P‐Co_3_O_4_ as both the cathode and anode was constructed to evaluate its overall efficacy as a bifunctional catalyst under alkaline conditions. Figure 4e shows that urea electrolysis has a considerable advantage over water electrolysis in hydrogen production. The urea electrolysis system required 400 mV less voltage than the water electrolysis system to achieve a current density of 50 mA cm^−2.^ The La, P‐Co_3_O_4_║La, P‐Co_3_O_4_‐based urea electrolyzer achieves ≈19.4% higher energy efficiency for hydrogen production than the water electrolyzer. To assess the stability of the La, P‐Co_3_O_4_ catalyst, prolonged electrochemical evaluations were performed using LSV before and after a 72‐h stability assessment at a constant current density of 10 mA cm^−2^ (Figure 4f). After 72 h of continuous operation (Figure 4g), no significant variation in overpotential was observed based on the post‐stability LSV curve, demonstrating that the catalyst maintained its electrocatalytic efficacy over time. The catalyst exhibited remarkable longevity with no substantial decline in activity, demonstrating its exceptional stability at the applied current density. Nevertheless, commercial catalysts IrO_2_║Pt/C have constrained endurance, with significant deterioration seen after 40 h of CP operation. Figure 4h and Table S3 (Supporting Information) compare the efficacy of the La, P‐Co_3_O_4_ catalyst and previously reported urea oxidation catalysts, which require elevated potentials to achieve a current density of 10 mA cm^−2^.

### 2.4 In Situ Raman Analysis

**Figure 5:**
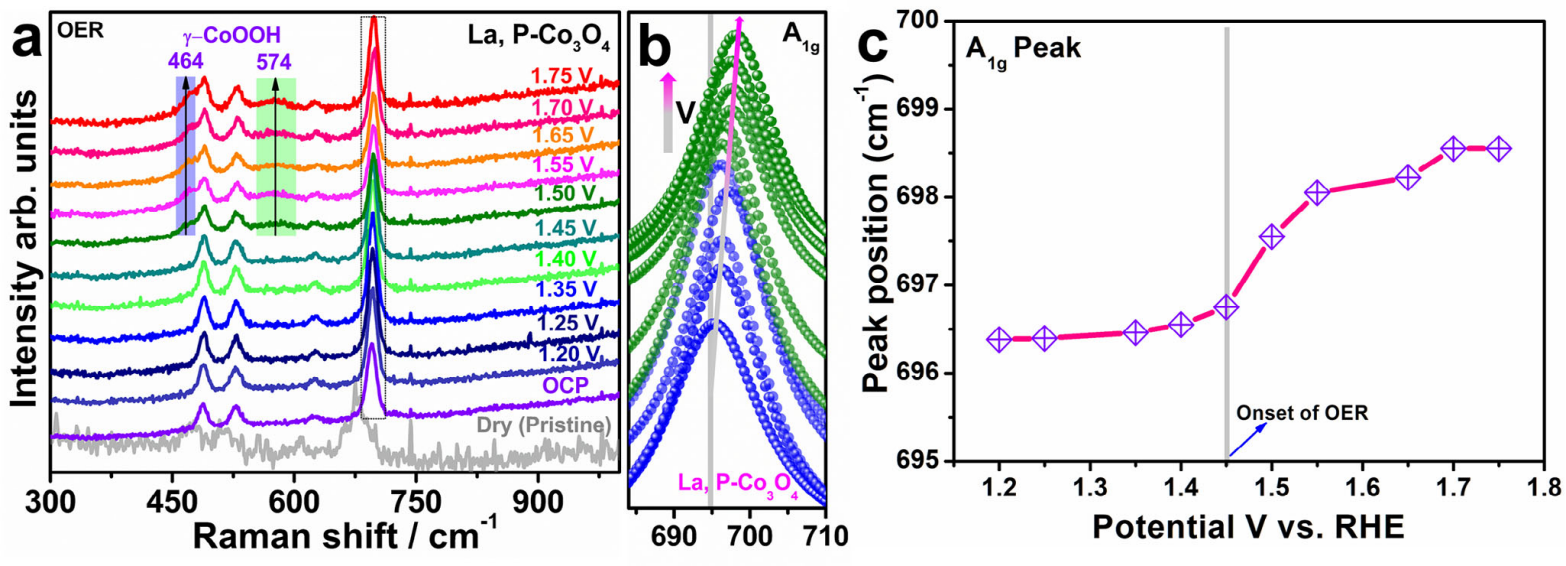
a) In situ Raman spectra of La, P‐Co_3_O_4_ at various constant potentials (1.20–1.75 V vs RHE). b) Analysis of Raman A_1g_ peaks of La, P‐Co_3_O_4_ based on Lorentzian function fitting (the gray vertical line indicates the actual A_1g_ position of the dry sample). c) Raman spectra of pristine Co_3_O_4_ and La, P‐Co_3_O_4_ before and after OER test.

In situ Raman spectroscopy experiments were conducted to monitor the correlations between the catalytic activity improvement, redox‐mediated surface reconstruction, and the altered local bonding environment in La, P‐Co_3_O_4_ under OER conditions (**Figure** **5**). As shown in Figure 5a, the peaks were preserved throughout the electrochemical tests, indicating that the basic spinel phase provides a robust scaffold for the hydroxylated amorphous phase above it. The dry sample exhibits four distinct Raman peaks (A_1g_, E_g_, and two F_2g_ modes), and the initial F_2g_ band at ≈200 cm^−1^ is absent owing to its overlap with a diffuse scattering of the electrolyte solution at low wavenumbers.^[^ ^54^ ^]^ These peaks are typical for the spinel phase.^[^ ^54^, ^55^ ^]^ For the La, P‐Co_3_O_4_ catalyst, an additional Raman signal appeared at ≈464 and 574 cm^−1^ as the applied potential increased from 1.50 to 1.75 V in CA mode. These peaks are ascribed to the formation of γ‐CoOOH species on the catalyst surface.^[^ ^56^, ^57^ ^]^ The γ‐CoOOH species was observed at 1.50 V, which is consistent with OER performance (Figure 5a). This result correlates with the durability of the γ‐CoOOH phase in an aqueous environment at 25 °C and pH 13, as indicated by the Pourbaix diagram.^[^ ^58^ ^]^ Notably, the γ‐CoOOH active sites are the dominant active phase and are more favorable to the outstanding OER activity of La, P‐Co_3_O_4_. Importantly, compared to β‐CoOOH, the conductivity of γ‐CoOOH is higher, taking advantage of its substantial interlayer distance (γ‐CoOOH = 6.8 Å), thereby enabling OH^−^ insertion.^[^ ^56^, ^59^, ^60^ ^]^ The shift and broadening of the A_1g_ band at 684 cm^−1^ indicate the oxidation of the cobalt (Co^3+^ → Co⁴^+^) on the electrocatalyst surface, as suggested by Jin et al.^[^ ^56^ ^]^ To gain deep insight into the progression of the local bonding conditions at the catalyst surface during the OER, the peak position, intensity, and full width at half maximum of the A_1g_ peak were obtained using Lorentzian function fitting (Figure 5b). The observed blue shift in Raman signals typically indicates lattice contraction and charge redistribution (Figure 5c). Under applied potential, the oxidation of Co^3^⁺ to Co⁴⁺ reduces the ionic radius, leading to Co─O bond shortening. This contraction increases the bond force constant, thereby raising the vibrational frequency and shifting the Raman peaks to higher wavenumbers.^[^ ^61^ ^]^ This improves OER activity by optimizing electronic structure, facilitating charge transfer, stabilizing reaction intermediates, and creating catalytically active surface defects like oxygen vacancies (Figure S11b, Supporting Information).

Pristine Co_3_O_4_ exhibits a blue shift in the A_1g_ mode under the same electrochemical conditions, indicating a degree of surface oxidation during the OER (Figure S12a, Supporting Information). Nonetheless, the magnitude of this change is much less than that seen for La, P‐Co_3_O_4_ (Figure S12b, Supporting Information), suggesting a more constrained lattice response and diminished redox flexibility. Furthermore, no new Raman peaks indicative of γ‐CoOOH production are seen in the pristine sample, suggesting a lack of significant surface reconstruction or dynamic phase development during electrochemical operation. This difference highlights the essential function of La and P co‐doping in modulating the structural and electrical characteristics of Co_3_O_4_. The increased ionic radius of La^3^⁺ causes lattice strain and improves the flexibility of the oxygen sublattice, facilitating cobalt oxidation and structural rearrangement under anodic conditions. Simultaneously, the electron‐withdrawing characteristic of P promotes charge delocalization and the creation of oxygen vacancies, both of which accelerate the conversion to γ‐CoOOH. These dopants jointly reduce the barrier for surface reconstruction, stabilize Co⁴⁺ species, and enhance electronic conductivity under OER conditions. Conversely, La, P‐Co_3_O_4_ exhibits a more pronounced A_1g_ peak shift and much larger intensity, signifying augmented Co─O bond strength, elevated structural polarization, and an increased density of active sites. Thus, La, P‐Co_3_O_4_ demonstrates a more significant Raman shift and dynamic γ‐CoOOH formation, characteristics lacking in pure Co_3_O_4_, emphasizing the efficacy of dopant engineering in activating and modifying catalytic surfaces for improved OER performance.

## 3 Conclusion

In summary, the as‐prepared La, P‐Co_3_O_4_ catalyst exhibited exceptional electrocatalytic performance for both water and urea oxidation, emphasizing its potential for sustainable hydrogen production. This notable phenomenon arises from the combined effects of La and P co‐doping, which significantly increase the ECSA, create numerous O_v_, increase the density of active sites, and enhance electronic conductivity. These modifications successfully address the inherent limitations of pristine Co_3_O_4_ catalysts. The decreased onset potential for urea oxidation highlights the effectiveness of the synthesized catalyst in alternative oxidation pathways, thereby reinforcing its potential use in energy‐efficient hydrogen generation. The addition of La and P influences the electronic structure and establishes a more advantageous local bonding environment, thereby promoting the oxidation of Co^3^⁺ to catalytically active Co⁴⁺ species while inhibiting charge accumulation during electrochemical processes. This facilitates a more advantageous reaction pathway by passing the potential‐determining redox step in Co_3_O_4_, resulting in dynamic surface reconstruction and improved OER and UOR kinetics. These insights improve the comprehension of defect engineering and dopant‐induced reconstruction in spinel oxides and offer a promising approach for the design of next‐generation catalysts for energy conversion and electrochemical devices.

## 4 Experimental Section

### Chemicals and Materials

Cobalt (II) nitrate hexahydrate (Co(NO_3_)_2_, 97%), 2‐methylimidazole (C_4_H_6_N_2_, 98%), lanthanum (III) nitrate hexahydrate (La(NO_3_)_2_, 97%), sodium hypophosphite (NaH_2_PO_2,_ 96%), urea (CO(NH_2_)_2_, 99%), potassium hydroxide (KOH, 85%), methanol (CH_3_OH, ≈99.99%), and ethanol (C_2_H_5_OH, 94%) were purchased from Daejung chemicals. Carbon cloth (CC), used as a working electrode substrate, was purchased from NARA Cell Tech Corporation. All chemicals were used without further purification. The catalyst substrate (CC) was also commercially acquired from NARA Cell Tech Corporation.

### Synthesis of ZIF‐67/La‐ZIF‐67

To synthesize ZIF‐67, clear solutions of Co(NO_3_)_2_.6H_2_O and 2‐methylimidazole were prepared by dissolving them separately in 100 mL of methanol. Next, the 2‐methylimidazole solution was added to the Co(NO_3_)_2_.6H_2_O solution, and the mixed solution was stirred at ambient temperature for 24 h. Following the completion of the reaction, the resulting purple solution was centrifuged, washed multiple times with methanol, and subsequently dried in an oven at 60 °C overnight. 0.1 g of the as‐synthesized ZIF‐67 powder was initially dispersed in 40 mL of a mixed solvent comprising 50% ethanol and 50% deionized water, and 0.1 g of La(NO_3_)_2_.6H_2_O was added with magnetic stirring for 4 h at ambient temperature. The etched ZIF‐67 particles were recovered by centrifugation, followed by three times washing with ethanol and deionized water. The particles were then dried at 60 °C in a vacuum oven overnight.

### Synthesis of Co_3_O_4_, La‐Co_3_O_4_, P‐Co_3_O_4_, and La, P‐Co_3_O_4_

To obtain Co_3_O_4_, ZIF‐67 was placed in a furnace and heated to 350 °C at a ramp rate of 2 °C min^−1^ in Ar gas flow for 2 h. A similar process was used to produce La‐Co_3_O_4_ by placing La‐ZIF‐67 in the furnace. To synthesize P‐Co_3_O_4_ and La and P‐Co_3_O_4_, NaH_2_PO_2_ (500 mg) on the upstream side, ZIF‐67 and La‐ZIF‐67 were placed in the downstream of a porcelain boat, respectively, and heated under the same conditions as above to achieve phosphorization.

## Conflict of Interest

The authors declare no conflict of interest.

## Author Contributions

B.A. performed conceptualization, investigation, methodology, and wrote the original draft. P.M. performed the software and methodology. R.K.D. performed the investigation, reviewed, and edited. R.V. and K.A. performed visualization. C.L.C. performed software. T.H.O. performed the visualization and reviewed. C.L.D. performed resources, reviewed, and edited. S.C.K. performed project administration, resources, wrote, reviewed, and edited.

## Supporting information



Supporting Information

## Data Availability

The data that support the findings of this study are available from the corresponding author upon reasonable request.
