# Supplementary material for: Unraveling Surface Reconstruction of MOF‐Derived La, P‐Co3O4 for Energy‐Efficient Water and Urea Electrolysis
Source: Small Methods. 2025 Sep 4;10(3):2500938. doi: 10.1002/smtd.202500938 (PMC12893293; doi:10.1002/smtd.202500938)
Supplement: Supplementary file 1 — Supporting Information [file SMTD-10-2500938-s001.docx]

Supplementary Information

Unraveling Surface Reconstruction of MOF-derived La, P-Co_3_O_4_ for Energy-Efficient Water and Urea Electrolysis

*Bharathi Arumugam,^a^* *Pandian Mannu,^b,c^ Ranjith Kumar Darman,^a^* *Ramkumar Vanaraj,^a^* *Krishnapandi Alagumalai,^a^ Chi-Liang Chen,^c^ Tae Hwan Oh,**^a^ Chung-Li Dong,*^b^ Seong-Cheol Kim*^a^*

*^a^ Department of Chemical Engineering, Yeungnam University, Gyeongsan, 38541, Korea*

*^b^ Department of Physics, Tamkang University, New Taipei City 25137, Taiwan*

*^c^National Synchrotron Radiation Rsearch Center, Hsinchu 30076, Taiwan*

**Corresponding authors:*

*C.L.D (*[*cldong@mail.tku.edu.tw*](mailto:cldong@mail.tku.edu.tw)*)*

*S.C.K (*[*sckim07@ynu.ac.kr*](mailto:sckim07@ynu.ac.kr)*)*

^#^*Equal contribution*

**Table of Contents**

Characterization

Electrochemical parameters

1. **Figure S1**. Field-emission scanning electron microscopy images of (a) ZIF-67 (b) La-ZIF-67 (c) Co_3_O_4_ (d) P-Co_3_O_4_ (e) La-Co_3_O_4_ and (f) La, P-Co_3_O_4_.
2. **Figure S2.** X-ray diffraction pattern of ZIF-67 and La-ZIF-67.
3. **Figure S3.** XPS survey spectra of Co_3_O_4_ and La, P-Co_3_O_4_.
4. **Figure S4.** (a) Soft-XAS spectra at Co L-edge; Deconvoluted Co L-edge spectra of (b) Co_3_O_4_ and (c) La, P-Co_3_O_4_.
5. **Figure S5.** Electrochemical impedance spectroscopy (a) HER. (b) UOR.
6. **Figure S6.** Cyclic voltammetry of all catalysts in the non-faradic region for OER (a) ZIF-67, (b) La-ZIF-67, (c) Co_3_O_4,_ (d) P-Co_3_O_4,_ (e) La-Co_3_O_4,_ (f) La, P-Co_3_O_4_ (g) C_dl_ values (h) ECSA values.
7. **Figure S7.** Cyclic voltammetry of all catalysts in the non-faradic region for HER: (a) ZIF-67, (b) La-ZIF-67, (c) Co_3_O_4,_ (d) P-Co_3_O_4,_ (e) La-Co_3_O_4,_ (f) La, P-Co_3_O_4,_ (g) C_dl_ values, (h) ECSA values.
8. **Figure S8.** Cyclic voltammetry of all catalysts in the non-faradic region for UOR (a) ZIF-67, (b) La-ZIF-67, (c) Co_3_O_4,_ (d) P-Co_3_O_4,_ (e) La-Co_3_O_4,_ (f) La, P-Co_3_O_4,_ (g) C_dl_ values, (h) ECSA values.
9. **Figure S9.** Post-catalysis of FESEM image of (a,b) Pristine Co_3_O_4_, and (c,d) La-Co_3_O_4_.
10. **Figure S10.** Post-catalysis Raman analyses of Co_3_O_4_ and La, P-Co_3_O_4_ before and after stability testing.
11. **Figure S11.** Post-catalysis XPS characterization. High-resolution XPS spectra of La, P-Co_3_O_4_ after a 48-hour long-term test. (a) Co 2p, (b) O 1s, (c) La 3d, and (d) P 2p.
12. **Figure S12.** (a) *In situ* Raman spectra of pristine Co_3_O_4_ at various constant potentials (1.20–1.60 V vs. RHE). (b) Analysis of Raman A_1g_ peaks of pristine Co_3_O_4_ based on Lorentzian function fitting (the gray vertical line indicates the actual A_1g_ position of the dry sample).
13. **Figure S13.** Schematic representation of the OER mechanism in alkaline environments.
14. **Table S1.** Oxygen evolution reaction’s electrochemical performance comparison between La, P-Co_3_O_4,_ and other Co_3_O_4_-based catalysts.
15. **Table S2.** Hydrogen evolution reaction’s electrochemical performance comparison between La, P-Co_3_O_4,_ and other Co_3_O_4_-based catalysts.
16. **Table S3.** Comparative analysis of the UOR activity La, P-Co_3_O_4_ (+) and La, P-Co_3_O_4_ (-) against other reported catalysts in urea-assisted water splitting in a two-electrode alkaline urea electrolyzer setup.

**Characterization**

All synthesized catalysts were initially examined for their crystal structure, purity, and phase using powder X-ray diffraction (PXRD, PANlytical X’Pert PRO, Cu Kα radiation). The microstructures and morphologies of the samples were examined using field emission scanning electron microscopy (FESEM, HITACHI S-4800), and their nanostructure morphologies were assessed using high-resolution transmission electron microscopy (HRTEM, Philips Tecnai 20). The chemical surface compositions and electronic properties of the catalysts were characterized using XPS (Thermo Scientific K-α surface analysis). XPS peak fitting and quantitative analysis were performed using XPSPEAK 4.1 software, which employs Gaussian/Lorentzian peak forms. Inductively coupled plasma optical emission spectroscopy was used to determine the weight percentage of elements using a Perkin Elmer OPTIMA 8300 spectrometer. All Raman tests were conducted using a Raman spectrometer (Model - UniDRON) with a Rayleigh excitation wavelength of 457 nm, covering a range of 200–2000 cm^−1^. The reliability and reproducibility of the obtained spectra were ensured by calibrating the Raman shift using a silicon reference sample (520.5 cm^−1^).

**Operando/*In situ* Raman Analysis**

Operando/*in situ* Raman analysis was performed in a specialized electrochemical cell at ambient temperature under an inert atmosphere. All Raman tests were conducted using a Raman spectrometer (Model: UniDRON) with a Rayleigh wavelength of 457 nm, covering a range of 200–2000 cm^−1^. The Raman shift was calibrated using a silicon standard sample (520.5 cm^−1^) to ensure the reliability and reproducibility of the obtained spectra. Hg/HgO served as the reference electrode, and Pt served as the counter electrode. The synthesized La, P-Co_3_O_4_ coated on CC served as the working electrode. *In situ* Raman spectra were recorded using the chronoamperometry (CA) mode in the potential range of 1.2–1.75 V vs. RHE. The sample was immersed in the electrolyte (1 M KOH), and all air bubbles were eliminated from the cell by circulating the electrolyte. *In situ* Raman measurements were performed in CA mode (i-t) at each specified voltage for 6 min to collect data.

**Electrochemical parameters**

The electrochemical parameters for the HER, OER, and UOR were conducted in a three-electrode cell using a Corrtest (CS350 in COM3) workstation. A platinum plate served as the counter electrode, and Hg/HgO served as the reference electrode in 1-M KOH (HER, OER) and 1-M KOH with 0.3-M urea (UOR) electrolyte. The working electrode was fabricated by grinding the catalyst with acetylene black and polyvinylidene fluoride at a ratio of 80:10:10. N-Methyl-2-pyrrolidone was used as the solvent. The catalyst ink was applied to CC (1 × 1 cm²) and dried at 60℃. The measured potentials were calibrated to a reversible hydrogen electrode (RHE) using the Nernst equation.

$$E_{\mathrm{RHE}}=E_{\left[ \left( Hg/HgO \right)\mathrm{or}\left( Ag/AgCl \right) \right]}+E_{\left[ \left( Hg/HgO \right)\mathrm{or}\left( Ag/AgCl \right) \right]}^{º}+0.59*pH+0.098$$

Before the electrochemical study, 50 cycles of CV were performed to stabilize the sample. A linear sweep voltammogram (LSV) test was conducted at a scan rate of 5 mV s⁻¹ to minimize the capacitive contribution. All current densities shown in this study are in the geometric surface area. The double-layer capacitance (C_dl_) and ECSA were determined using CV at scan speeds ranging from 10 to 100 mV s⁻¹ in the non-faradic region.

$$ECSA=R_{f}S=\frac{C_{dl}}{C_{s}},$$

where Cs denotes the specific capacitance, and the units for Rf and S are cm^2^_real_cm^−2^_geo_ and cm^2^_geo,_ respectively; S represents the geometric surface area of the smooth metal electrode, which is generally equal to the geometric area of the CC electrode (S = 1 cm^2^). EIS measurements were performed at an AC amplitude of 10 mV across a frequency range of 100 kHz–0.1 Hz. The long-term stability of the electrocatalyst was evaluated by CP tests at constant overpotential. Overall water splitting and urea oxidation were achieved by using the La, P-Co_3_O_4_ electrocatalyst as both the anode and cathode in a dual-cell configuration.

**Figure S1.** Field-emission scanning electron microscopy images of (a) ZIF-67, (b) La-ZIF-67, (c) Co_3_O_4,_ (d) P-Co_3_O_4,_ (e) La-Co_3_O_4,_ and (f) La, P-Co_3_O_4_.


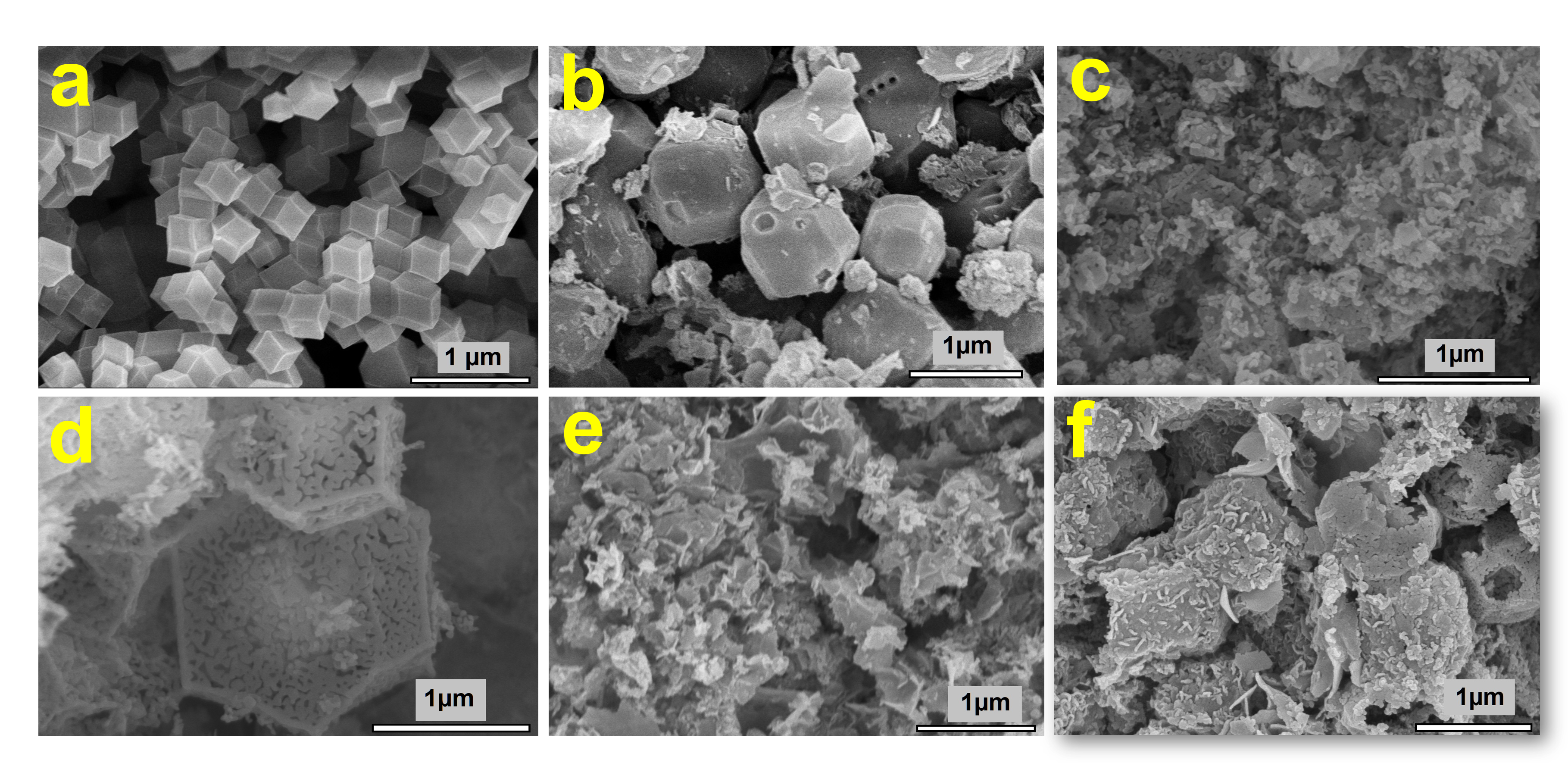

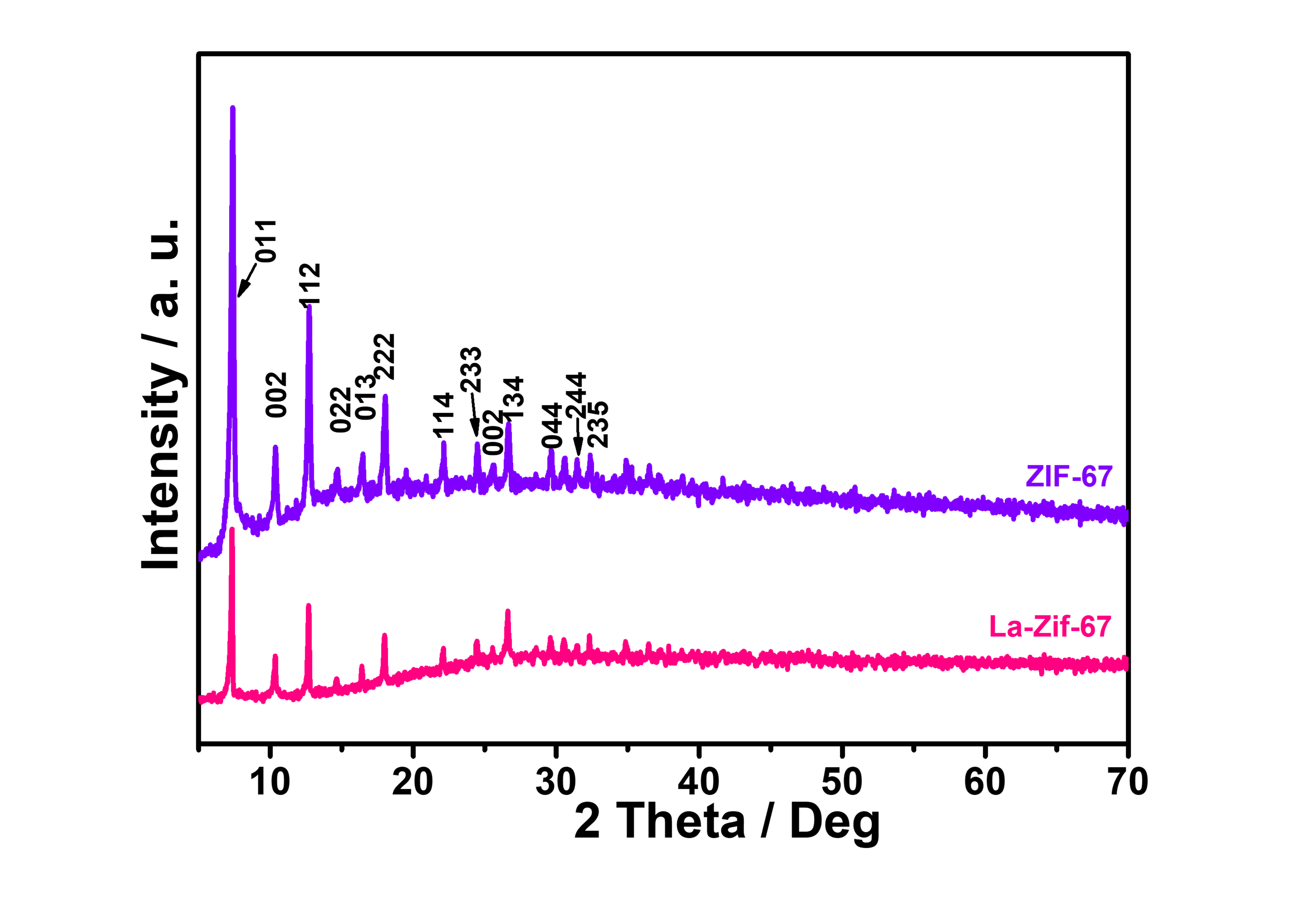


**Figure S2.** X-ray diffraction pattern of ZIF-67 and La-ZIF-67.





**Figure S3.** XPS survey spectra of Co_3_O_4_ and La, P-Co_3_O_4_.

**Figure S4.** (a) Soft-XAS spectra at Co L-edge; Deconvoluted Co L-edge spectra of (b) Co_3_O_4_ and (c) La, P-Co_3_O_4_.


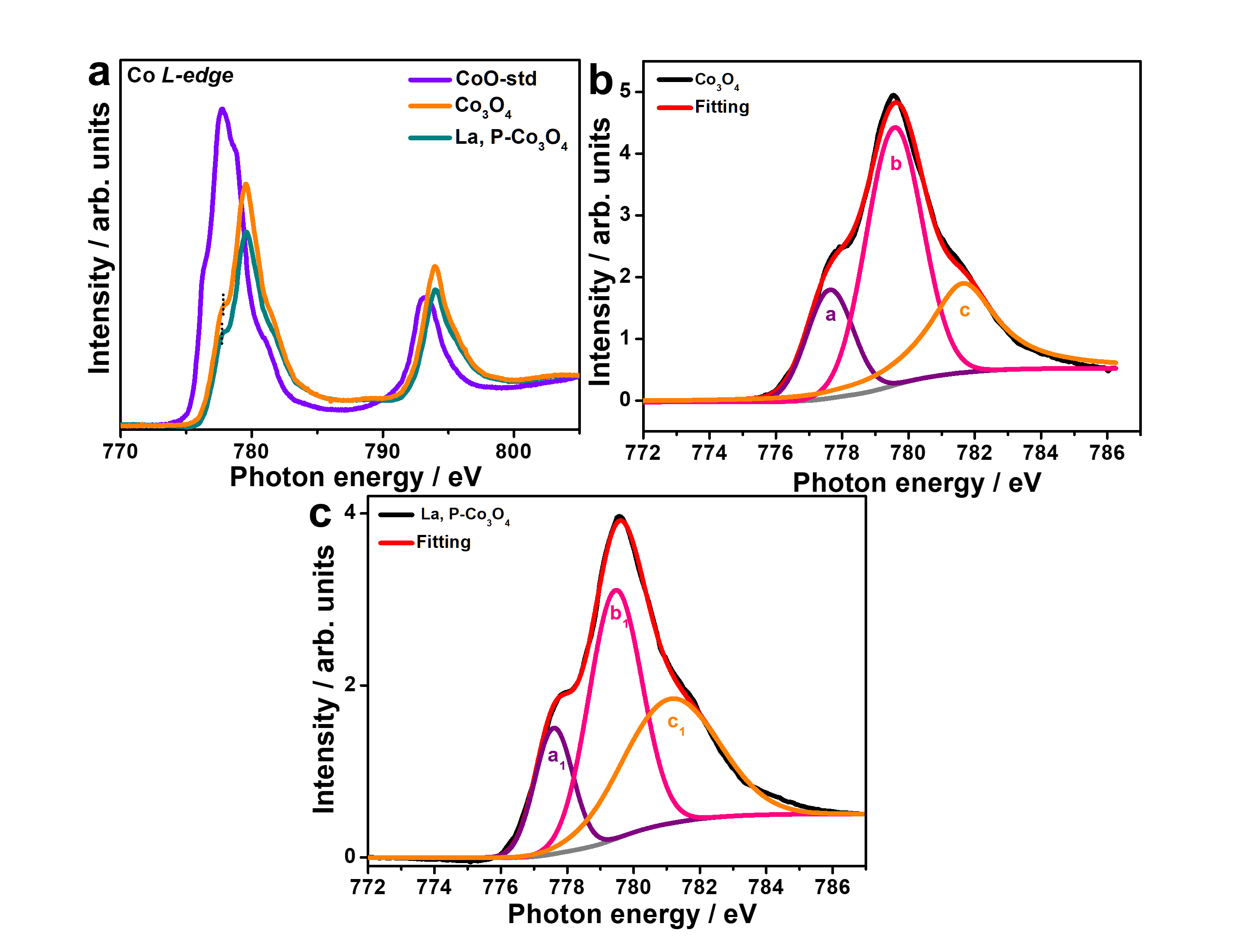

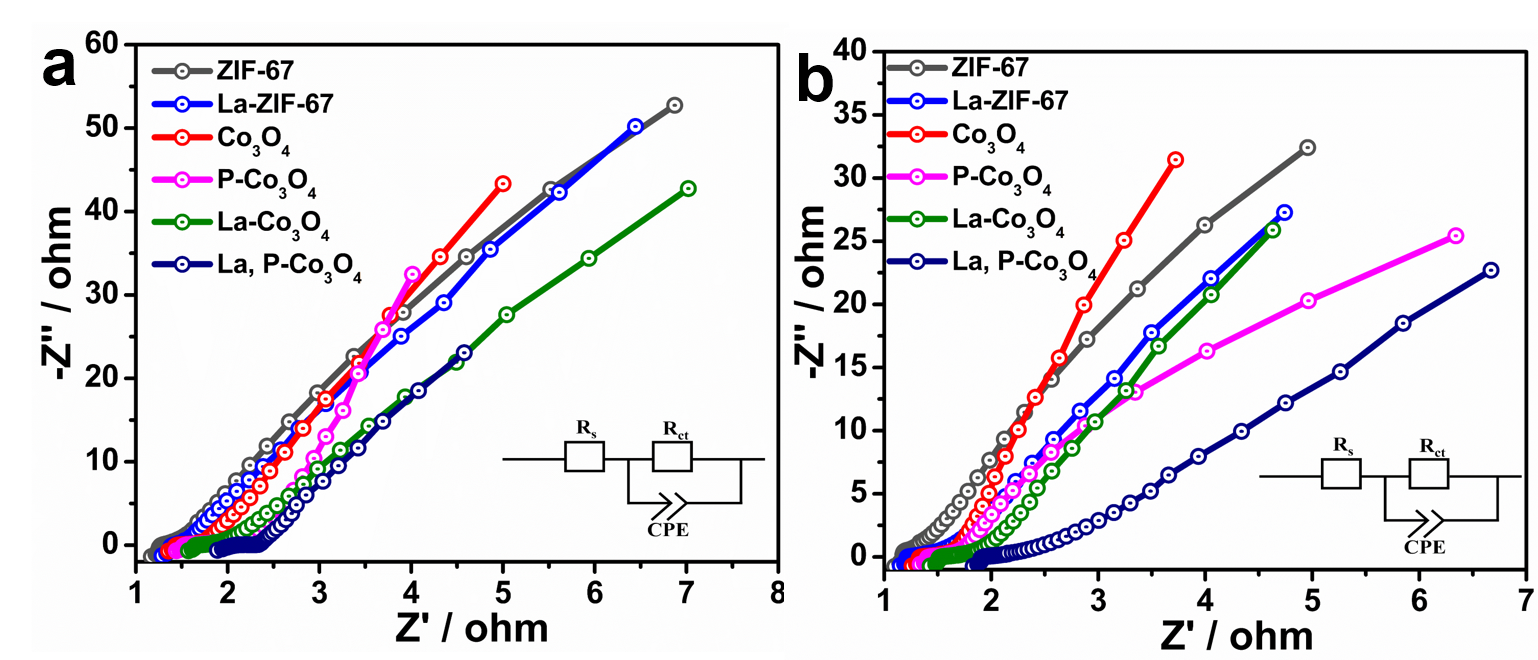


**Figure S5.** Electrochemical impedance spectroscopy for (a) HER. (b) UOR.

**Figure S6.** Cyclic voltammetry of all catalysts in the non-faradic region for OER (a) ZIF-67, (b) La-ZIF-67, (c) Co_3_O_4,_ (d) P-Co_3_O_4,_ (e) La-Co_3_O_4,_ (f) La, P-Co_3_O_4,_ (g) C_dl_ values, (h) ECSA values.


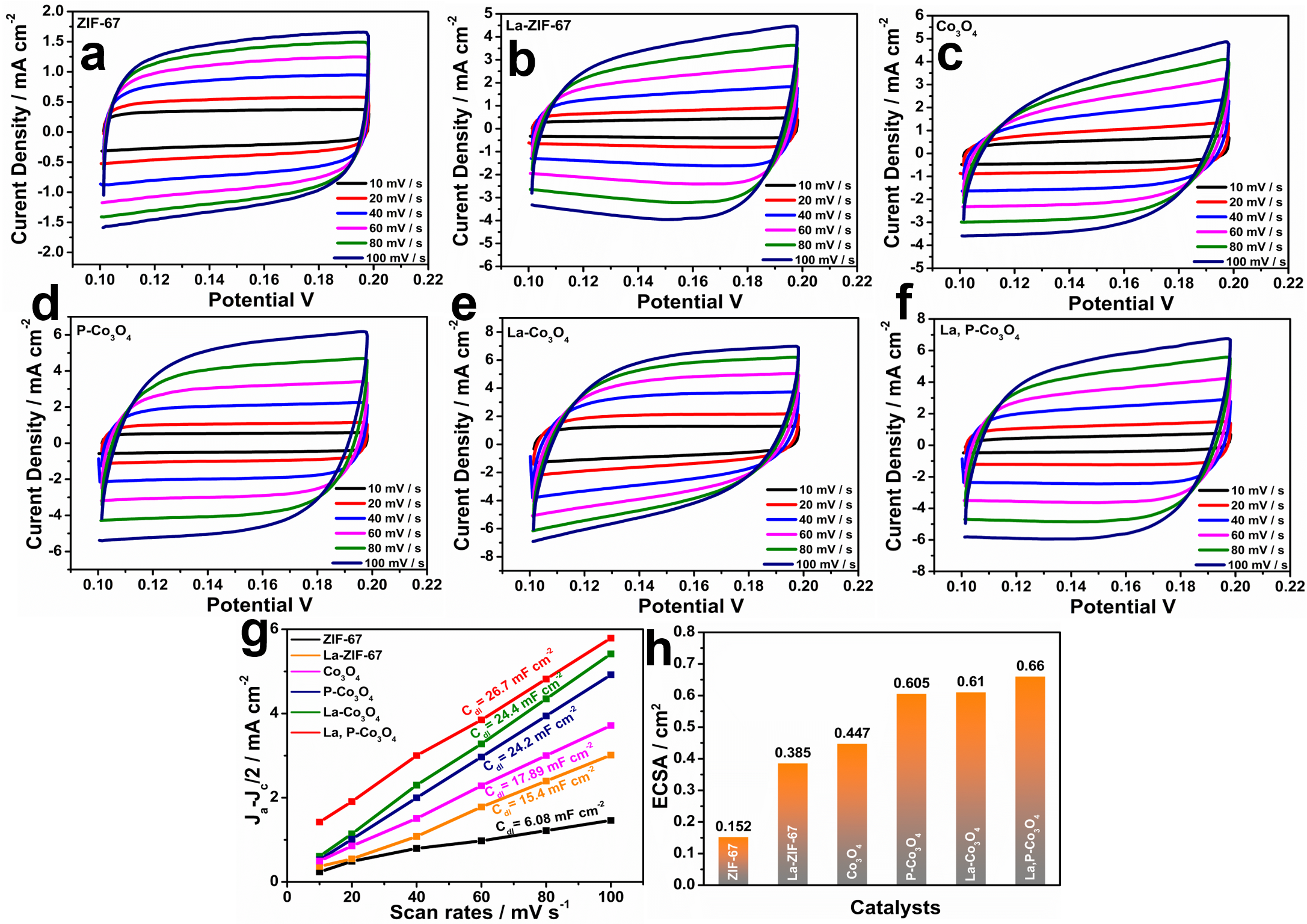


**Figure S7.** Cyclic voltammetry of all catalysts in the non-faradic region for HER (a) ZIF-67, (b) La-ZIF-67, (c) Co_3_O_4,_ (d) P-Co_3_O_4,_ (e) La-Co_3_O_4,_ (f) La, P-Co_3_O_4,_ (g) C_dl_ values, (h) ECSA values.


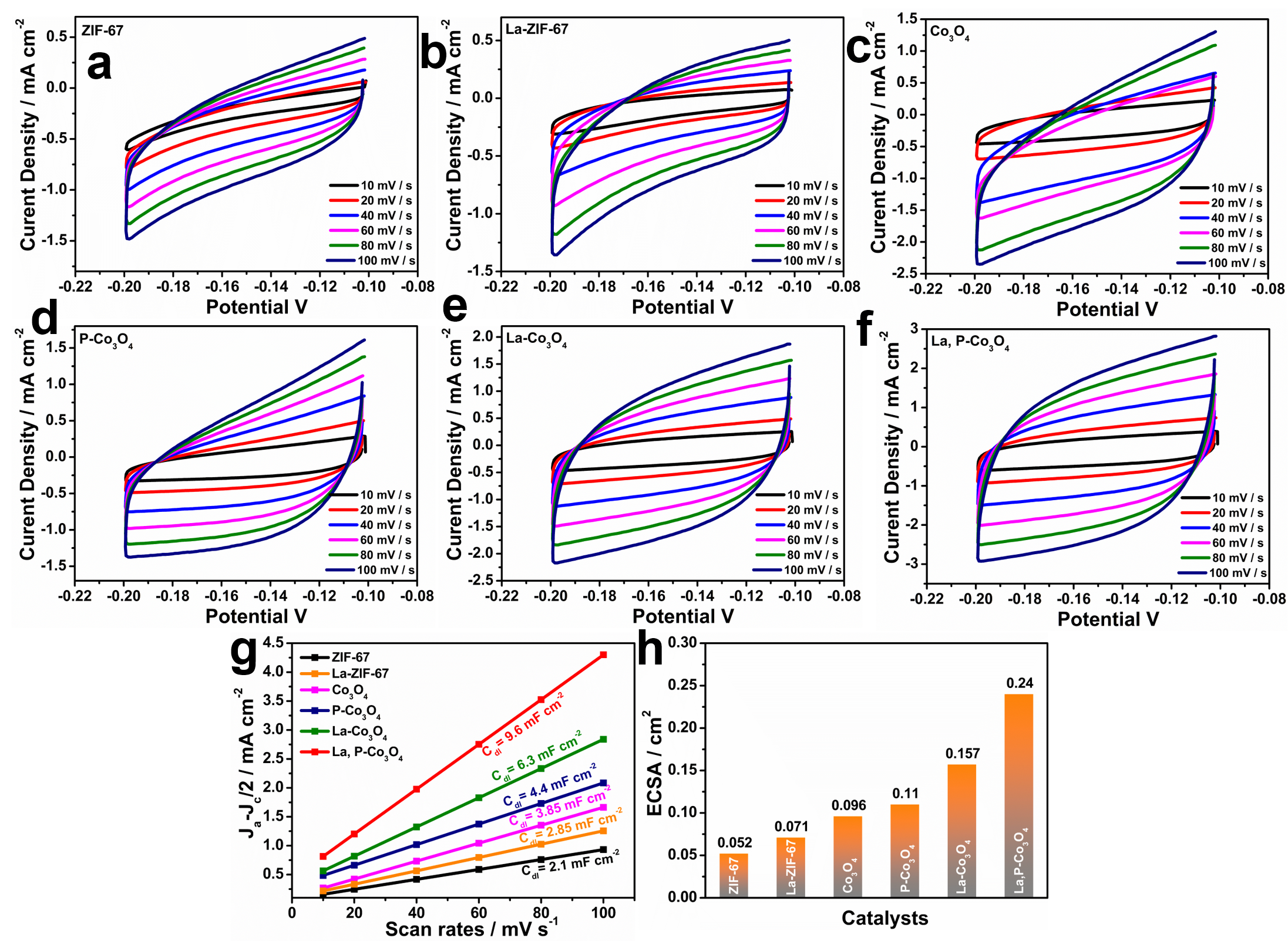


**Figure S8.** Cyclic voltammetry of all catalysts in the non-faradic region for UOR (a) ZIF-67, (b) La-ZIF-67, (c) Co_3_O_4,_ (d) P-Co_3_O_4,_ (e) La-Co_3_O_4,_ (f) La, P-Co_3_O_4,_ (g) C_dl_ values, (h) ECSA values.


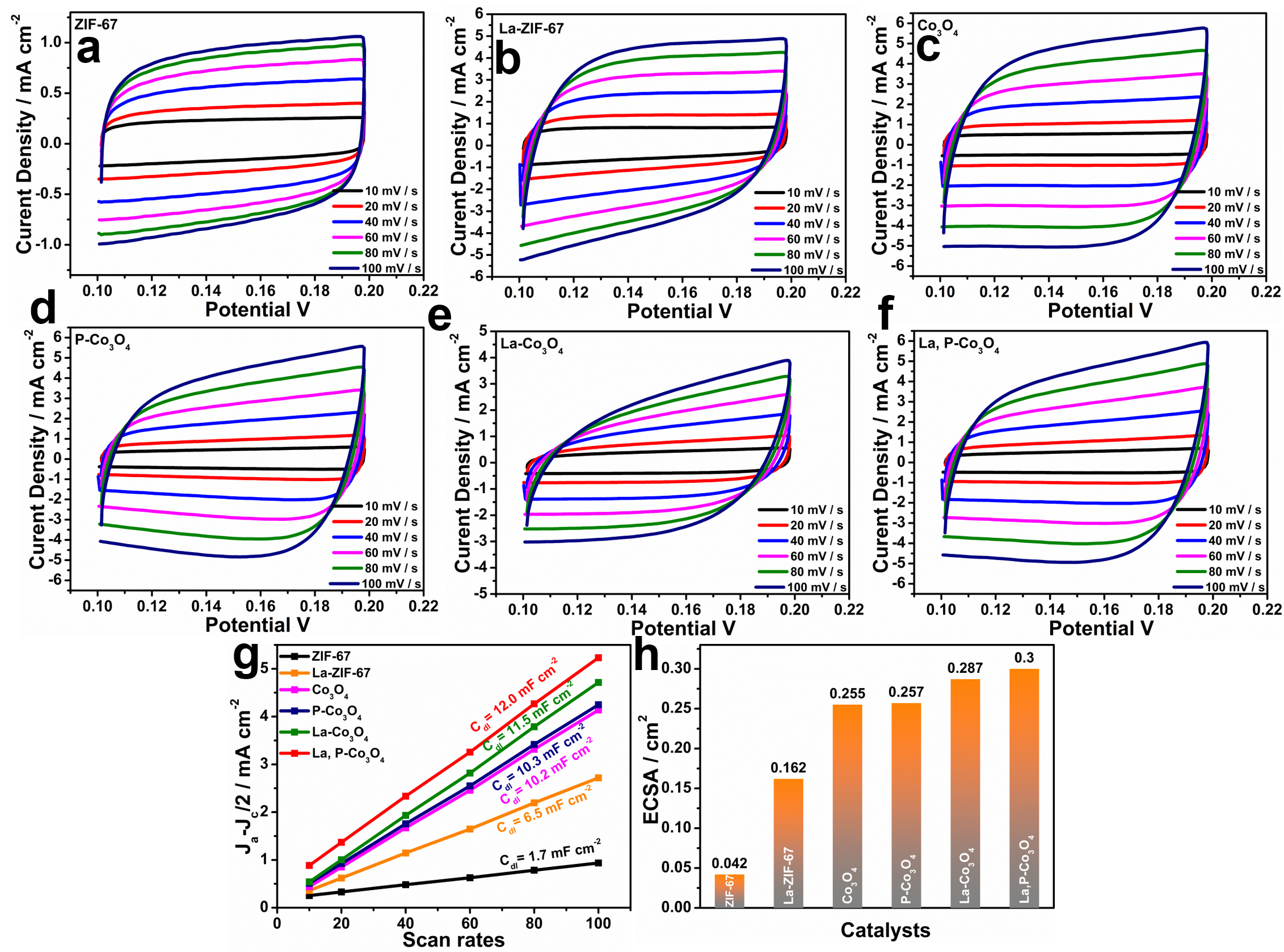

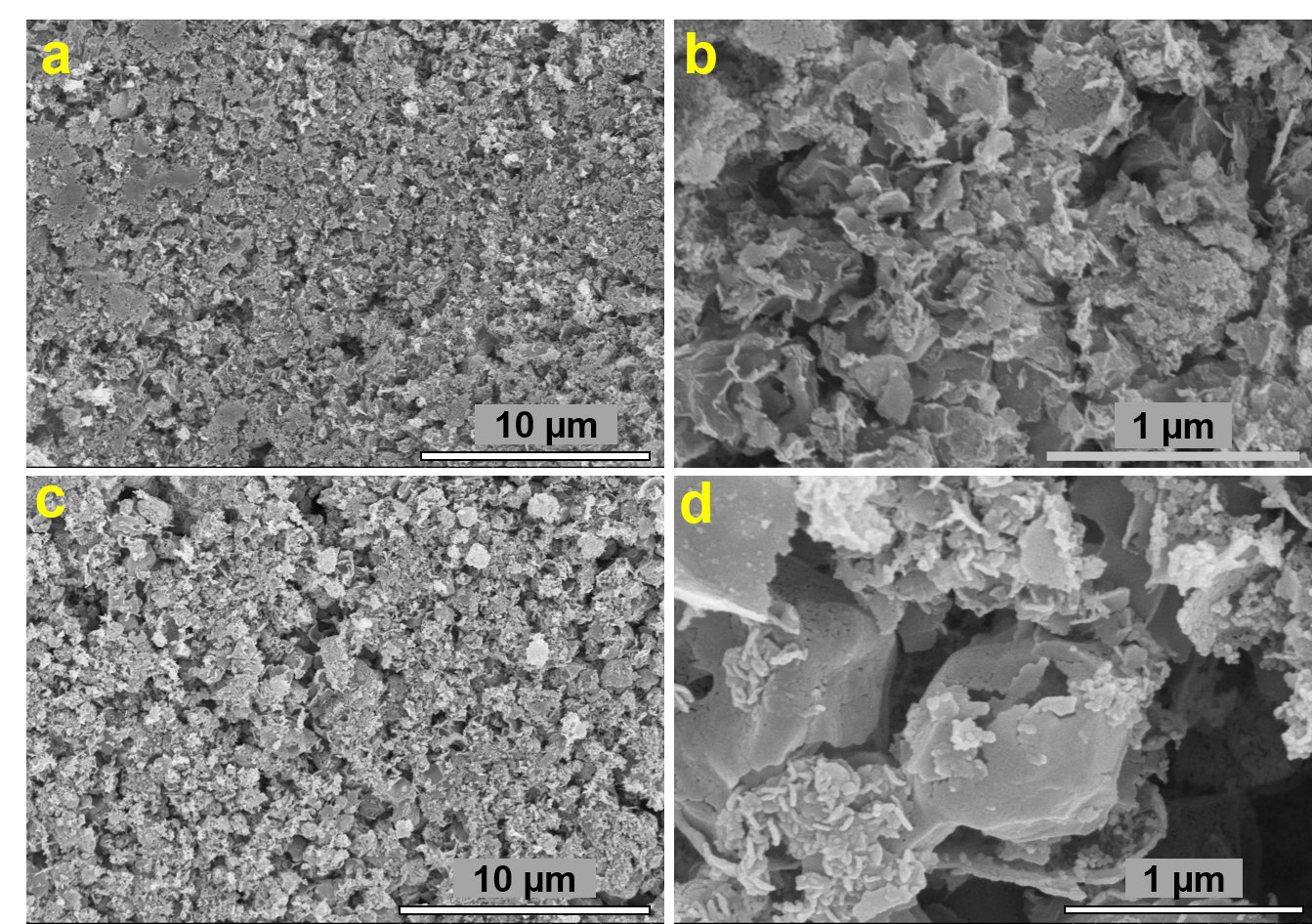


**Figure S9.** Post-catalysis FESEM image of (a, b) Pristine Co_3_O_4_ and (c, d) La, P-Co_3_O_4_.

**Figure S10.** Post-catalysis Raman analyses of Co_3_O_4_ and La, P-Co_3_O_4_ before and after stability testing.




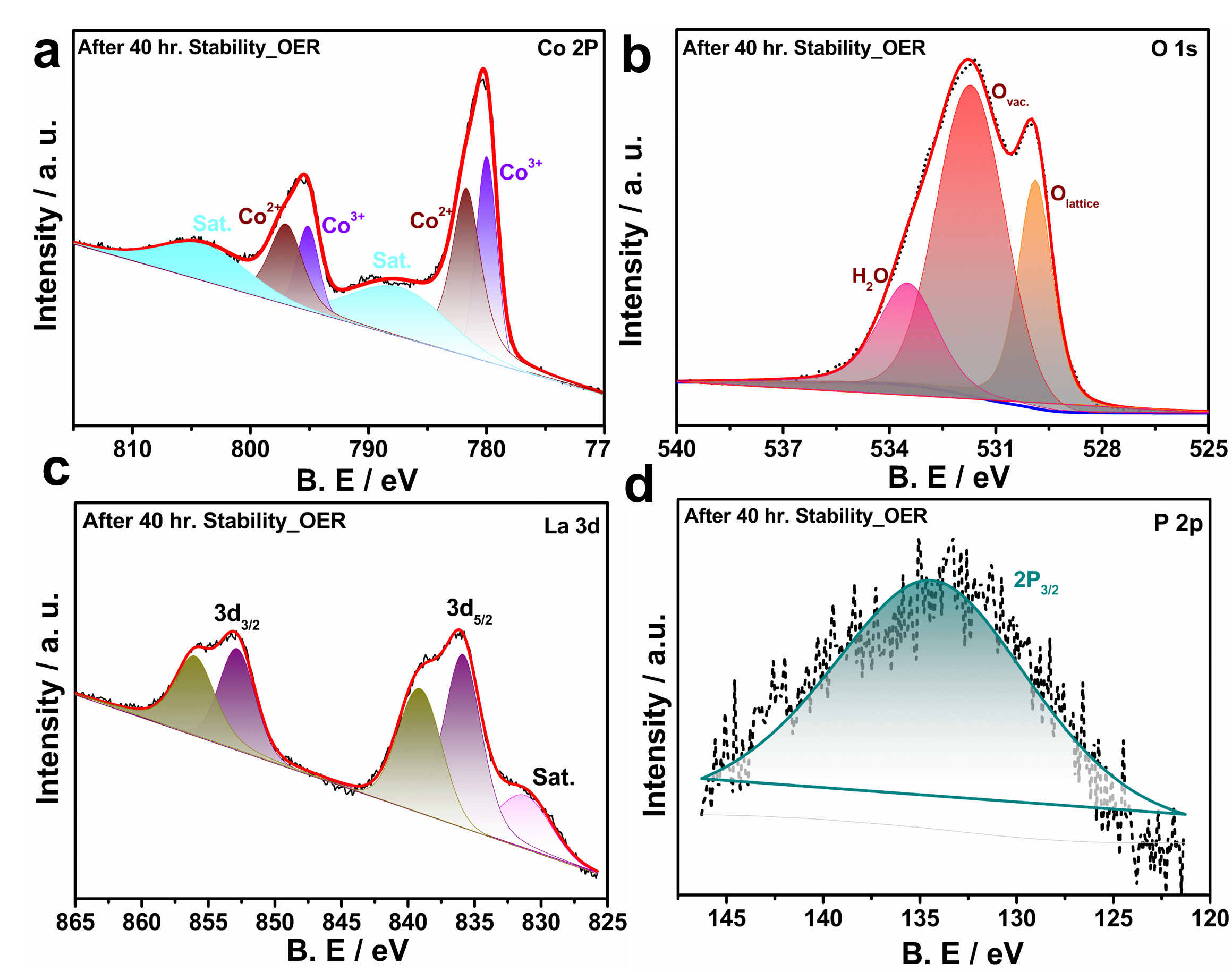


**Figure S11.** Post-catalysis XPS characterization. High-resolution XPS spectra of La, P-Co_3_O_4_ after a 48-hour long-term test. (a) Co 2p, (b) O 1s, (c) La 3d, and (d) P 2p.


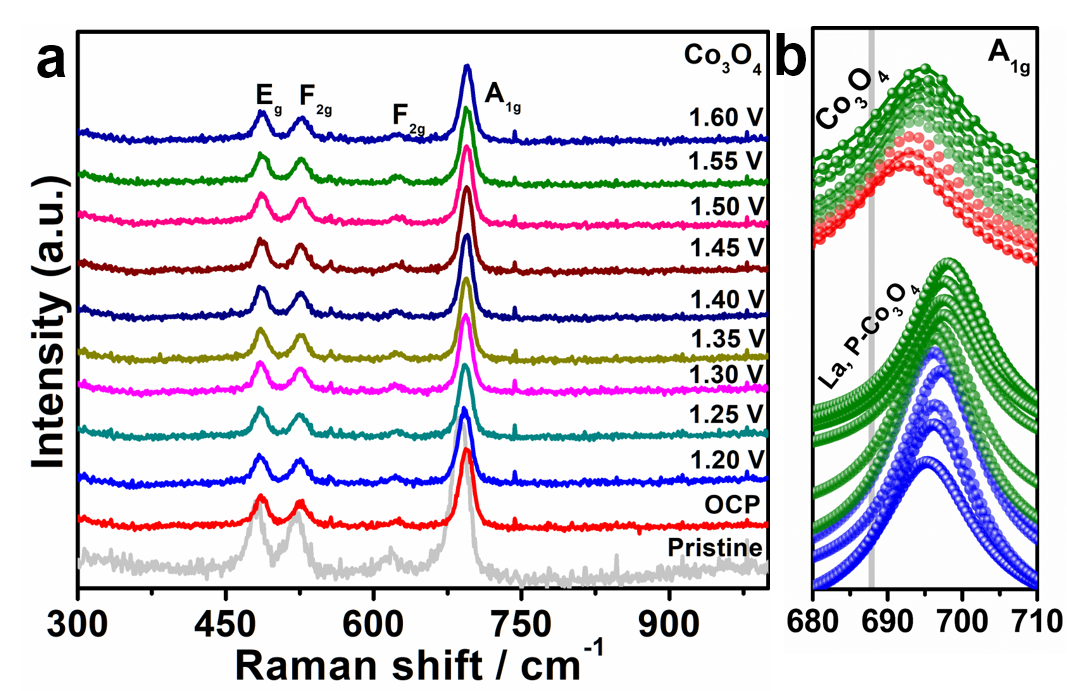


**Figure S12.** (a) *In situ* Raman spectra of pristine Co_3_O_4_ at various constant potentials (1.20–1.60 V vs. RHE). (b) Analysis of Raman A_1g_ peaks of pristine Co_3_O_4_ based on Lorentzian function fitting (the gray vertical line indicates the actual A_1g_ position of the dry sample).


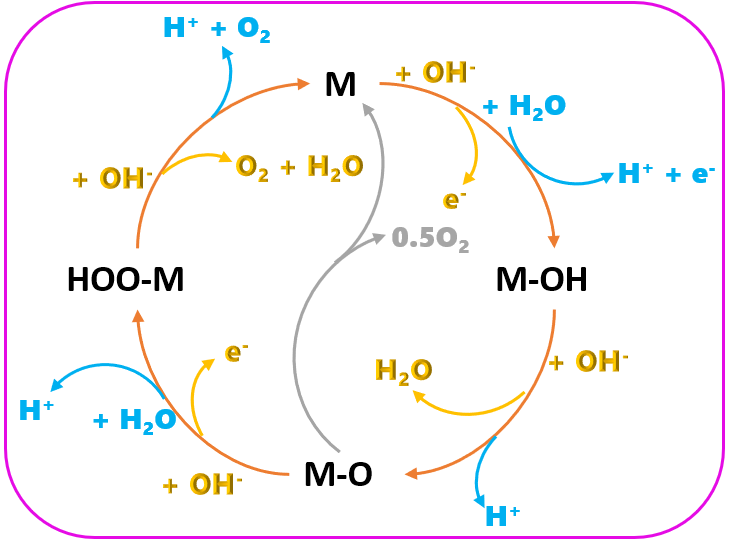


**Figure S13.** Schematic representation of the OER mechanism in alkaline environments.

The OER mechanism in alkaline medium is shown in **Figure 13**. The *insitu* Raman spectroscopy, ESR, and XPS analyses confirm the presence and functional importance of oxygen vacancies in Co_3_O₄, La-Co_3_O₄, and La, P-Co_3_O₄. Notably, the in-situ Raman spectra reveal an accelerated surface reconstruction to γ-CoOOH during OER in the La, P-Co_3_O₄ sample, revealing that O_v_ plays a crucial role in modulating the catalytic process. Consistent with our previous reports on O_v_-rich Co_3_O_4_ electrocatalysts, we propose that oxygen vacancies in La, P-Co_3_O_4_ promote pre-oxidation of Co^2+^ to Co^3+^ during the OER, which is essential for forming the active Co-OOH intermediate.^1–3^ These modifications facilitate charge transfer and enhance catalytic kinetics. Therefore, it is suggested that the presence of O_v_ on the surface of La, P-Co_3_O_4_ modulates the adsorption energy of OH^−^ at Co sites and acts as a key intermediary in the transformation of adsorbed OH^•^ into OOH^•^ species, thus catalyzing the OER.^4,5^ The oxygen defects on La, P-Co_3_O_4_ were more readily filled with OH^•^, facilitating the pre-oxidation of Co sites and supporting reconstruction/deprotonation of the M-OOH^•^ intermediate. Additionally, La³⁺ doping introduces lattice strain, which increases oxygen sublattice flexibility and promotes the oxidation of Co sites under anodic conditions. Simultaneously, the electron-withdrawing nature of P^5^⁺ further facilitates charge delocalization and the creation of oxygen vacancies, thereby accelerating the conversion to the active CoOOH phase. The ESR (**Figure 2j**) and XPS (**Figure 2c**) results further support the critical role of oxygen defects, showing enhanced density and lower Co oxidation states in La, P-Co_3_O_4_. Consequently, oxygen vacancies are essential in enhancing OER activity by improving OH^−^-adsorption, enabling Co oxidation, and promoting the surface reconstruction into catalytically active oxyhydroxide phases. These effects are synergistically amplified by La and P co-doping, resulting in an improved electronic structure and enhanced catalytic efficiency.

**Table S1:** Oxygen evolution reaction’s electrochemical performance comparison between La, P-Co_3_O_4,_ and other Co_3_O_4_-based catalysts.

| Catalysts | Overpotential  at 50 mA cm^−2^ (mV) | Tafel slope  (mV dec^−1^) | C_dl_  (mF cm^−2^) | ECSA  (cm^2^) |
| --- | --- | --- | --- | --- |
| Co_3_O_4_ | 400 | 50.4 | 17.9 | 0.44 |
| P- Co_3_O_4_ | 388 | 47.1 | 24.2 | 0.60 |
| La-Co_3_O_4_ | 371 | 44.3 | 24.4 | 0.61 |
| La, P-Co_3_O_4_ | **351** | **39.1** | **26.7** | **0.66** |

**Table S2:** Hydrogen evolution reaction’s electrochemical performance comparison between La, P-Co_3_O_4,_ and other Co_3_O_4_-based catalysts.

| Catalysts | Overpotential  at −50 mA cm^−2^ (mV) | Tafel slope  (mV dec^−1^) | C_dl_  (mF cm^−2^) | ECSA  (cm^2^) |
| --- | --- | --- | --- | --- |
| Co_3_O_4_ | 354 | 54.3 | 3.8 | 0.09 |
| P- Co_3_O_4_ | 322 | 47 | 4.4 | 0.11 |
| La-Co_3_O_4_ | 292 | 37.9 | 6.3 | 0.15 |
| La, P-Co_3_O_4_ | **222** | **28.1** | **9.6** | **0.24** |

**Table S3:** Comparative analysis of the UOR activity of La, P-Co_3_O_4_ under anodic (+) and cathodic (−) conditions against other reported catalysts in urea-assisted water splitting in a two-electrode alkaline urea electrolyzer setup.

| Catalysts | Urea Conc.(M) | Cell Potential (V) @ 10 mA cm^−2^ | Ref. |
| --- | --- | --- | --- |
| La, P-Co_3_O_4_ | **1 M KOH + 0.33 M** | **1.44** | **This Work** |
| Co_2_Mo_3_O_8_ | 1 M KOH + 0.5 M | 1.50 | ^6^ |
| CoMn/CoMn_2_O_4_ | 1 M KOH + 0.5 M | 1.51 | ^7^ |
| CoFeCr LDH/NF | 1 M KOH + 0.3 M | 1.50 | ^8^ |
| NC-FNCP | 1 M KOH + 0.5 M | 1.52 | ^9^ |
| Co_x_Mo_y_S-CC | 1 M KOH + 0.5 M | 1.50 | ^10^ |
| CoFe | 1 M KOH + 0.33 M | 1.47 | ^11^ |
| Rh-Co_3_S_4_/CoO_x_ NTs | 1 M KOH + 0.5 M | 1.45 | ^12^ |
| P-CoNi_2_S_4_ | 1 M KOH + 0.5 M | 1.55 | ^13^ |
| CoSx/Co-MOF | 1 M KOH + 0.5 M | 1.48 | ^14^ |
| CoS_2_ NA/Ti | 1 M KOH + 0.3 M | 1.59 | ^15^ |

**Reference**

(1) Xiao, Z.; Huang, Y. C.; Dong, C. L.; Xie, C.; Liu, Z.; Du, S.; Chen, W.; Yan, D.; Tao, L.; Shu, Z.; Zhang, G.; Duan, H.; Wang, Y.; Zou, Y.; Chen, R.; Wang, S. Operando Identification of the Dynamic Behavior of Oxygen Vacancy-Rich Co3O4for Oxygen Evolution Reaction. *J. Am. Chem. Soc.* **2020**, *142* (28), 12087–12095. https://doi.org/10.1021/jacs.0c00257.

(2) Huang, Y. C.; Chen, W.; Xiao, Z.; Hu, Z.; Lu, Y. R.; Chen, J. L.; Chen, C. L.; Lin, H. J.; Chen, C. Te; Arul, K. T.; Wang, S.; Dong, C. L.; Chou, W. C. In Situ/ Operando Soft X-Ray Spectroscopic Identification of a Co4+Intermediate in the Oxygen Evolution Reaction of Defective Co3O4Nanosheets. *J. Phys. Chem. Lett.* **2022**, *13* (35), 8386–8396. https://doi.org/10.1021/acs.jpclett.2c01557.

(3) Mannu, P.; Dharman, R. K.; Nga, T. T. T.; Mariappan, A.; Shao, Y. C.; Ishii, H.; Huang, Y. C.; Kandasami, A.; Oh, T. H.; Chou, W. C.; Chen, C. L.; Chen, J. L.; Dong, C. L. Tuning of Oxygen Vacancies in Co3O4 Electrocatalyst for Effectiveness in Urea Oxidation and Water Splitting. *Small* **2024**, *2403744*, 1–13. https://doi.org/10.1002/smll.202403744.

(4) Tao, H. B.; Fang, L.; Chen, J.; Yang, H. Bin; Gao, J.; Miao, J.; Chen, S.; Liu, B. Identification of Surface Reactivity Descriptor for Transition Metal Oxides in Oxygen Evolution Reaction. *J. Am. Chem. Soc.* **2016**, *138* (31), 9978–9985. https://doi.org/10.1021/jacs.6b05398.

(5) Grimaud, A.; Hong, W. T.; Shao-Horn, Y.; Tarascon, J. M. Anionic Redox Processes for Electrochemical Devices. *Nat. Mater.* **2016**, *15* (2), 121–126. https://doi.org/10.1038/nmat4551.

(6) Zhang, K.; Liu, C.; Graham, N.; Zhang, G.; Yu, W. Modulation of Dual Centers on Cobalt-Molybdenum Oxides Featuring Synergistic Effect of Intermediate Activation and Radical Mediator for Electrocatalytic Urea Splitting. *Nano Energy* **2021**, *87* (March). https://doi.org/10.1016/j.nanoen.2021.106217.

(7) Wang, C.; Lu, H.; Mao, Z.; Yan, C.; Shen, G.; Wang, X. Bimetal Schottky Heterojunction Boosting Energy-Saving Hydrogen Production from Alkaline Water via Urea Electrocatalysis. *Adv. Funct. Mater.* **2020**, *30* (21), 1–10. https://doi.org/10.1002/adfm.202000556.

(8) Wang, Z.; Liu, W.; Hu, Y.; Guan, M.; Xu, L.; Li, H.; Bao, J.; Li, H. Cr-Doped CoFe Layered Double Hydroxides: Highly Efficient and Robust Bifunctional Electrocatalyst for the Oxidation of Water and Urea. *Appl. Catal. B Environ.* **2020**, *272* (March). https://doi.org/10.1016/j.apcatb.2020.118959.

(9) Zhang, J.; Huang, S.; Ning, P.; Xin, P.; Chen, Z.; Wang, Q.; Uvdal, K.; Hu, Z. Nested Hollow Architectures of Nitrogen-Doped Carbon-Decorated Fe, Co, Ni-Based Phosphides for Boosting Water and Urea Electrolysis. *Nano Res.* **2022**, *15* (3), 1916–1925. https://doi.org/10.1007/s12274-021-3810-4.

(10) Li, P.; Zhuang, Z.; Du, C.; Xiang, D.; Zheng, F.; Zhang, Z.; Fang, Z.; Guo, J.; Zhu, S.; Chen, W. Insights into the Mo-Doping Effect on the Electrocatalytic Performance of Hierarchical CoxMoyS Nanosheet Arrays for Hydrogen Generation and Urea Oxidation. *ACS Appl. Mater. Interfaces* **2020**, *12* (36), 40194–40203. https://doi.org/10.1021/acsami.0c06716.

(11) Province, L. Co 3 O 4 纳米立方体的可控合成及其 CO 氧化反应性能 Morphology-Controlled Synthesis of Co 3 O 4 Nanocubes And. **2014**, *30* (2), 382–388.

(12) Nguyen, D. C.; Doan, T. L. L.; Prabhakaran, S.; Kim, D. H.; Kim, N. H.; Lee, J. H. Rh Single Atoms/Clusters Confined in Metal Sulfide/Oxide Nanotubes as Advanced Multifunctional Catalysts for Green and Energy-Saving Hydrogen Productions. *Appl. Catal. B Environ.* **2022**, *313* (February). https://doi.org/10.1016/j.apcatb.2022.121430.

(13) Lu, X. F.; Zhang, S. L.; Sim, W. L.; Gao, S.; Lou, X. W. Phosphorized CoNi2S4 Yolk-Shell Spheres for Highly Efficient Hydrogen Production via Water and Urea Electrolysis. *Angew. Chemie - Int. Ed.* **2021**, *60* (42), 22885–22891. https://doi.org/10.1002/anie.202108563.

(14) Xu, H.; Ye, K.; Zhu, K.; Yin, J.; Yan, J.; Wang, G.; Cao, D. Template-Directed Assembly of Urchin-like CoS: X/Co-MOF as an Efficient Bifunctional Electrocatalyst for Overall Water and Urea Electrolysis. *Inorg. Chem. Front.* **2020**, *7* (14), 2602–2610. https://doi.org/10.1039/d0qi00408a.

(15) Wei, S.; Wang, X.; Wang, J.; Sun, X.; Cui, L.; Yang, W.; Zheng, Y.; Liu, J. CoS2 Nanoneedle Array on Ti Mesh: A Stable and Efficient Bifunctional Electrocatalyst for Urea-Assisted Electrolytic Hydrogen Production. *Electrochim. Acta* **2017**, *246*, 776–782. https://doi.org/10.1016/j.electacta.2017.06.068.
